# Monitoring the Spatiotemporal Dynamics of Invasive *Pedicularis kansuensis* in Bayinbuluke Alpine Wetlands: A Novel Spectral Index Framework Using PlanetScope Time Series (2021–2025)

**DOI:** 10.3390/plants15050806

**Published:** 2026-03-06

**Authors:** Enzhao Zhu, Alim Samat, Wenbo Li, Kaiyue Luo

**Affiliations:** 1 State Key Laboratory of Ecological Safety and Sustainable Development in Arid Lands, Xinjiang Institute of Ecology and Geography, Chinese Academy of Sciences, Urumqi 830011, China; zhuenzhao22@mails.ucas.ac.cn; 2 University of Chinese Academy of Sciences, Beijing 100049, China; 3 China-Kazakhstan Joint Laboratory for Remote Sensing Technology and Application, Al-Farabi Kazakh National University, Almaty 050012, Kazakhstan; 4 Xinjiang Key Laboratory of RS & GIS Application, Urumqi 830011, China; 5 Department of Electrical, Computer and Biomedical Engineering, University of Pavia, 27100 Pavia, Italy; wenbo.li01@universitadipavia.it; 6 College of Surveying and Geoinformatics, Tongji University, Shanghai 200070, China; luokaiyue@tongji.edu.cn

**Keywords:** invasive plants, alpine wetlands, spectral index, PlanetScope, *Pedicularis kansuensis*

## Abstract

The expansion of the invasive species *Pedicularis kansuensis* threatens the ecological integrity of alpine wetlands, particularly in the Bayinbuluke, northwestern China. However, operational monitoring remains challenging. Conventional indices often lack specificity in heterogeneous alpine backgrounds, while deep learning models are typically too data-intensive to support consistent, multi-year mapping. To develop a rapid, reliable, and operational method for monitoring this invader, we proposed a novel, species-specific spectral index, the *Pedicularis kansuensis* Index (PKI), using the blue, green, and red-edge bands of high-resolution (3 m) PlanetScope imagery. The PKI constructs a robust target signal by integrating distinct spectral features derived from in situ hyperspectral measurement with a grayscale morphological opening (GrMO) refinement to suppress background noise. A comprehensive validation against seven established benchmarks indices (e.g., NDVI, RI, and ARI) demonstrated the superior performance of PKI across the central alpine wetlands of Bayinbuluke (2841 km^2^). It achieved the highest separability with an M-statistic of 1.36. Furthermore, the index attained an overall accuracy of 93.52% (95% CI: 92.3–94.7%), and an F1-score of 93.28% (95% CI: 92.0–94.5%), effectively minimizing confusion with co-occurring native vegetation and background. Applying this framework to a five-year time series (2021–2025) revealed a distinct cycle of outbreaks and relaxation. Specifically, the invaded area increased to 2168 ha in 2022, then decreased to 160 ha in 2025. Spatial analysis further identified stable invasion hotspots of 161.6 ha, highlighting key targets for long-term containment. Meanwhile, 94.4% of the invaded area was transient, lasting only one year (4824.7 ha). These results confirm that the PKI is a physically interpretable, accurate, and computationally efficient tool for monitoring invasive species in heterogeneous alpine environments. It facilitates timely and targeted ecosystem management.

## 1. Introduction

Alpine wetlands are functionally pivotal yet highly vulnerable ecosystems in high-mountain regions [1]. By buffering seasonal water storage and release [2], maintaining long-term soil carbon pools [2], and sustaining specialized biota [3], they underpin downstream water security and biodiversity across many highland regions [4]. Despite their importance, these ecosystems are increasingly vulnerable to rapid degradation under the combined pressures of climate warming and intensifying human disturbance [5]. For example, alpine wetlands in China’s Sanjiangyuan Region have contracted by 29% over recent decades [6], and those in the First Meander of the Yellow River are facing multiple ecological stresses, including widespread grassland degradation, desertification and wetland loss [7,8]. Although glacial retreat and permafrost thaw have received significant attention, biological invasion is emerging as a less noticeable but rapidly accelerating stressor that can restructure plant communities, disrupt nutrient cycling, and degrade ecosystem function [9]. In high-altitude environments where recovery is intrinsically slow, invasive expansion can push wetlands across the ecological thresholds, transforming heterogeneous, functional landscapes into simplified and degraded ones [10,11]. Therefore, accurately and robustly invasion dynamics monitoring is essential for timely intervention and sustaining alpine wetland.

This challenge is particularly acute in the Bayinbuluke Grassland (42°47′ N, 84°09′ E), a key ecological barrier in arid northwestern China. There, the invasive species *Pedicularis kansuensis* Maxim [12] has expanded rapidly, contributing to pronounced grassland degradation [13,14]. Through potential allelopathic effects and competitive superiority in light acquisition, *P. kansuensis* becomes the dominant species and excludes other plants [15]. Its low palatability further strengthens grazing-mediated feedback, facilitating the spread in disturbed and overgrazed areas [16]. Although local authorities have undertaken mowing-based control, field operations are often inefficient over such large, inaccessible grasslands due to a lack of spatially explicit guidance.

Although traditional field surveys provide reliable invasion information, they are labor-intensive, costly, and inherently spatially discontinuous. This limits their practicality across the vast, topographically complex Bayinbuluke Grassland [17,18]. Remote sensing offers a scalable alternative, providing synoptic and repeatable observations of large and often inaccessible landscapes. This enables consistent mapping of invasion patterns over space and time [19]. However, detecting *P. kansuensis* remains challenging because invaded areas are typically mixed with native species, and occur within highly heterogeneous alpine wetland backgrounds.

Recent advances in machine learning (ML) and deep learning (DL) have achieved impressive accuracy in invasive species monitoring [20,21]. For example, Dao et al. mapped invasive species in a heterogeneous grassland ecosystem in southern Ontario, Canada, using a random forest classifier. Chen et al. detected small patches of the invasive *Spartina alterniflora* in the Yellow River Delta, China, using super-resolution technique and evolution analysis-based image segmentation. However, these approaches often require large, year-specific training datasets [10,18], particularly for DL, where limited training data can lead to a marked decline in model performance [22,23,24]. Model performance can also degrade when transferred across years with shifting phenology and background conditions [10]. Additionally, the complexity of the models and their computational demands may hinder their routine operational adoption by conservation practitioners. Conversely, vegetation indices offer a straightforward and interpretable method to summarize essential spectral properties and have been extensively employed for rapid, large-scale vegetation monitoring [25,26,27]. However, conventional indices such as NDVI and EVI are not designed to discriminate the spectral characteristics of a specific plant species, which limits their utility for species-level invasion monitoring [28]. Consequently, a simple, efficient, and directly applicable remote sensing index for monitoring *P. kansuensis* is still lacking.

To address this gap, this study introduces the *Pedicularis kansuensis* Index (PKI), which integrates in situ plant spectral measurements with the bands of PlanetScope imagery. The PKI highlights the spectral characteristics of *P. kansuensis* and reduces interference from heterogeneous alpine wetland backgrounds, thereby improving separability in mixed-canopy conditions. Specifically, the study aims to: (1) analyze the spectral characteristics of *P. kansuensis* and co-occurring native species, (2) develop and validate PKI for accurate *P. kansuensis* mapping at 3 m spatial resolution, and (3) reconstruct the spatiotemporal invasion dynamics of the Bayinbuluke wetlands from 2021 to 2025.

## 2. Results

### 2.1. The Performance of PKI

The effectiveness of PKI for discriminating *P. kansuensis* from other land-cover and vegetation types was evaluated through a systematic benchmark against seven commonly used indices (RI, NDVI, NDRE, GNDVI, CIRE, CIG, and ARI). All indices (see Section 5.4.1) were computed from the same atmospherically corrected PlanetScope surface reflectance imagery and assessed using an identical reference dataset, ensuring sensor-consistent and method-consistent comparison. Results are reported as a coherent evidence chain from spatial expression to sample-level distributions and, finally, a quantitative separability metric, thereby linking interpretability with statistical support.

#### 2.1.1. Comparative Spatial Performance of PKI and Benchmark Indices

**Figure 1:**
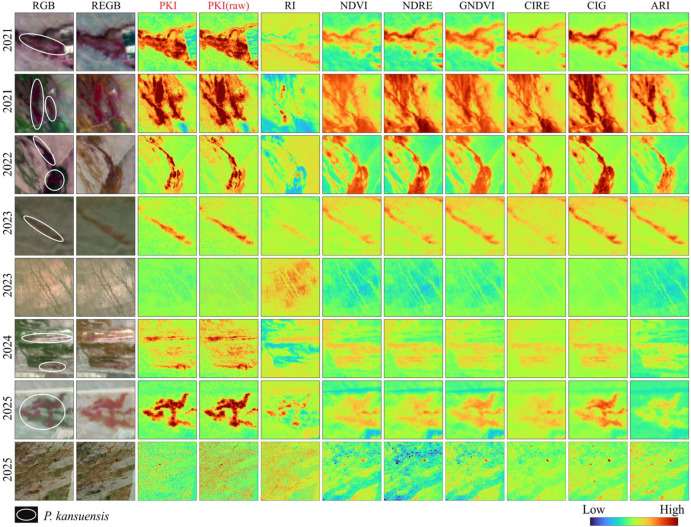
Multi-temporal visual comparison of the proposed PKI against seven reference indices (RI, NDVI, NDRE, GNDVI, CIRE, CIG, and ARI) for *P. kansuensis* monitoring. For each row, the first two columns show the corresponding RGB composite and REGB composite (Red edge-Green-Blue), followed by index maps. The white circles in the first row indicate the *P. kansuensis* areas.

Figure 1 compares the spatial distribution of the proposed PKI and the benchmark indices. Across acquisition dates, PKI produced the clearest spatial contrast between *P. kansuensis* and surrounding backgrounds. The response over *P. kansuensis* patches was compact and spatially coherent, while the surrounding matrix remained comparatively low and homogeneous, which is critical for delineation in heterogeneous alpine grassland-wetland mosaics. In mixed-canopy settings, this behavior indicates that the index response is aligned with the targeted flowering-related spectral signature while remaining less sensitive to non-target variability.

In comparison, greenness-oriented indices (NDVI, GNDVI) primarily tracked overall vegetation vigor, high responses were not confined to *P. kansuensis* and frequently extended into vigorous co-occurring native vegetation, reducing specificity. Red-edge/chlorophyll-sensitive indices (NDRE, CIRE, CIG, and RI) improved contrast in some scenes but often responded strongly to healthy non-target canopies and background gradients, leading to less stable patch boundaries where *P. kansuensis* co-occurred with productive grassland communities. ARI, although pigment-oriented, showed greater scene dependence and spatial variability in heterogeneous backgrounds, resulting in less consistent delineation across years.

The effect of the refinement step was then assessed by contrasting PKI with PKI (raw). Because *P. kansuensis* typically forms dense and spatially homogeneous patches, the GrMO refinement introduced minimal change within target patches, preserving both their intensity and geometry. The main change occurred in the surrounding background, where spectrally similar but structurally heterogeneous land covers (e.g., mixed grass components and partially exposed soil) were suppressed. This spatial filtering reduced speckle-like fluctuations and sharpened patch-to-background transitions, yielding cleaner surrounding matrices while retaining the target signal.

#### 2.1.2. Sample-Level Separability of PKI Versus Benchmark Indices

**Figure 2:**
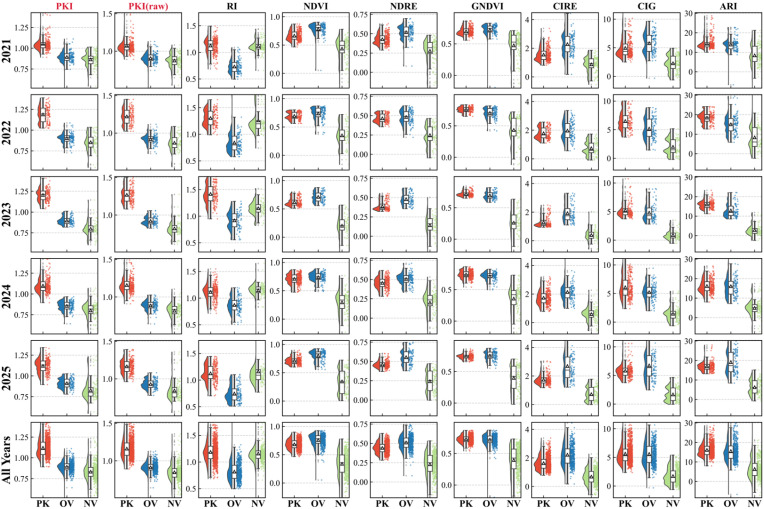
The sample-level distributions of the proposed indices (PKI and PKI (raw)) and seven benchmark indices (RI, NDVI, NDRE, GNDVI, CIRE, CIG, and ARI) for three classes: PK (*P. kansuensis*, red), OV (other vegetation, blue), and NV (non-vegetation, green).

To assess separability across land-cover types, we computed PKI for all validation samples (Figure 2), including *P. kansuensis* (PK), other vegetation (OV), and non-vegetation (NV). PKI showed the clearest separation of PK from OV and NV, with compact within-class spread and minimal overlap, consistently across 2021–2025, indicating robustness to background variation and imaging conditions.

In contrast, benchmark indices showed substantial overlap with the background. Greenness-based indices (e.g., NDVI, GNDVI) mainly tracked biomass and became indistinguishable from invasive patches at peak growth. Red/red-edge and pigment-related indices (e.g., NDRE, CIRE, CIG, RI) provided only limited improvement and remained sensitive to background cover. ARI separated vegetation from NV but could not reliably distinguish *P. kansuensis* from co-occurring native species.

Finally, spatial refinement was important. PKI(raw) enhanced the target signal but, as a pixel-wise index, was more affected by noise and fine-scale background variability, producing occasional high values in OV and NV. Applying grayscale morphological opening (GrMO) reduced these spatially incoherent fluctuations while preserving the coherent signal of aggregated *P. kansuensis* patches, thereby improving separability.

#### 2.1.3. Quantitative Separability Using the M-Statistic

**Figure 3:**
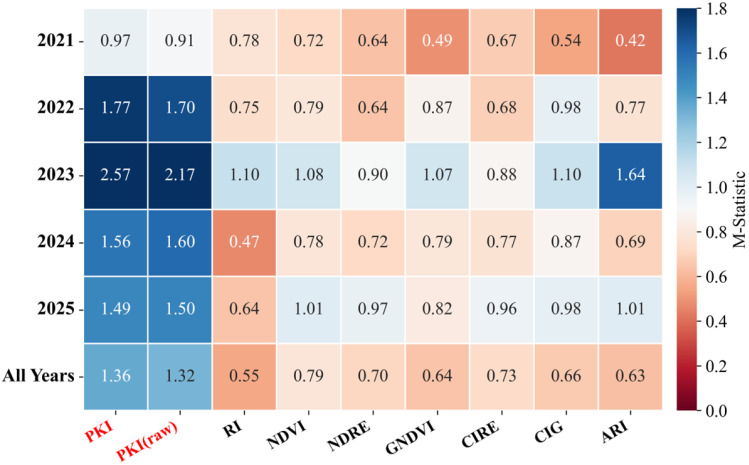
The M-statistic quantifying separability between *P. kansuensis* and background classes for the proposed indices (PKI and PKI (raw)) and seven benchmark indices (RI, NDVI, NDRE, GNDVI, CIRE, CIG, and ARI).

To quantify the effectiveness of various indices in distinguishing *P. kansuensis*, we employed M-statistics on the validation dataset (Figure 3) to assess the separability between *P. kansuensis* (PK) and other classes (OV and NV). The M-statistic provides a compact quantification of separability, and the resulting values consistently align with both the observed spatial performance and the sample-level distributions (Figure 2). The results show that PKI achieved the highest overall separability, with an average M-value of 1.36 over five years. Its unrefined version, PKI (raw), followed with an average M-value of 1.32. All benchmark indices remained below the conventional M > 1.0 criterion in the multi-year average results (RI: 0.55, NDVI: 0.79, NDRE: 0.70, GNDVI: 0.64, CIRE: 0.73, CIG: 0.66 and ARI: 0.63), indicating substantial overlap between the classes. These results confirm that PKI consistently exhibits a larger distributional gap between *P. kansuensis* and non-target samples than existing indices do.

The interannual M-statistics further support the robustness of the PKI. From 2022 to 2025, PKI exceeded the separability criterion in every year (1.77, 2.57, 1.56, and 1.49, respectively) and ranked highest among all indices. The peak separability of PKI occurred in 2023 (2.57), while the strongest baseline competitor that year was ARI (1.64). And most other indices clustered near 1.10 or below. In 2021, all indices fell below 1.0, indicating an intrinsically challenging separation scenario. Nevertheless, PKI produced the highest value (0.97) and remained closest to the threshold.

Finally, the quantitative results of PKI and PKI (raw) confirm the refinement mechanism. While both variants consistently ranked first and second, PKI achieved the best average separability over five years. This suggests that GrMO primarily enhances robustness and performance by suppressing and stabilizing heterogeneous background responses. This reduces the overlap in the value distributions of PKI between different classes.

### 2.2. Accuracy Assessment and Analysis

**Figure 4:**
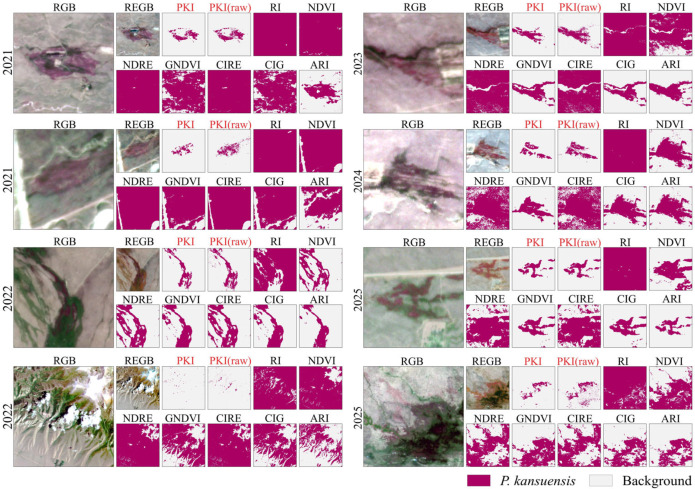
Visual comparison of binary extraction results for *P. kansuensis* across years using the proposed indices (PKI and PKI (raw)) and benchmark indices (RI, NDVI, NDRE, GNDVI, CIRE, CIG, and ARI). For each case, the corresponding RGB and REGB (red edge-green-blue) composites are shown alongside the thresholded maps.

To compare the classification performance of all indices under a consistent and operational setting, all continuous index images were converted into binary *P. kansuensis* maps (Figure 4) and evaluated using Accuracy, F1-score, Cohen’s kappa, Precision, and Recall (Table 1 and Table 2). Because PKI and PKI (raw) were specifically formulated to yield an interpretable one-sided response, they were segmented using a single fixed rule (PKI > 1). For the benchmark indices, however, no physically meaningful universal threshold can be specified a priori, and their value ranges vary across indices and years. Therefore, benchmark indices were segmented using a semi-automatic threshold range based on the boxplot whiskers of validation dataset (Figure 2), which avoids manual tuning while accommodating index- and year-dependent value ranges (Section S1).

#### 2.2.1. Performance of *P. kansuensis* Classification Using PKI Versus Benchmark Indices

Across 2021–2025, PKI consistently achieved the strongest classification performance (Table 1 and Table 2), confirming that the separability advantages established in Section 2.1 are retained after binarization. In the All-Years evaluation, PKI reached 93.52% Accuracy (95% CI: 92.3–94.7%), 93.28% F1-score (95% CI: 92.0–94.5%), and 87.03% kappa (95% CI: 84.6–89.4%) in average, clearly exceeding both PKI (raw) (91.38%, 90.83%, 82.73%). By comparison, the benchmark indices performed markedly worse: their Accuracy ranged from 57.31% to 68.79%, and their kappa values remained low (15.58–38.11%), indicating weak agreement beyond chance. Importantly, the advantage is not limited to a single year. PKI achieved 95.63% Accuracy (95% CI: 92.5–98.0%) in 2022, peaked at 98.80% in 2023 (95% CI: 97.2–100.0%), and remained high in 2024 (96.27%, 95% CI: 94.3–98.0%) and 2025 (92.05%, 95% CI: 89.5–94.6%). Even in 2021, which appears to be the most challenging year for all methods, PKI maintained the highest Accuracy (87.82%, 95% CI: 84.3–90.9%) and a comparatively strong kappa (75.77%, 95% CI: 69.1–81.6%), indicating that the method still provides meaningful discrimination when background conditions compress separability.

Performance differences among indices were primarily expressed through their error balance. Under semi-automatic threshold segmentation, benchmark indices generally produced very high Recall (96.14–100%), but at the cost of low Precision (53.69–61.26%) in All-Years average (Table 1 and Table 2). This combination indicates pervasive commission errors, i.e., extensive over-detection where non-target pixels fall within the accepted interval (Figure 4). Such behavior is consistent with the broad sensitivity of conventional indices: greenness indices (NDVI, GNDVI) respond strongly to vigorous co-occurring native vegetation, while red-edge/chlorophyll-sensitive indices (NDRE, CIRE, CIG, and RI) remain influenced by chlorophyll and canopy structure variability that is not unique to *P. kansuensis*. In contrast, PKI achieved a markedly more favorable trade-off, with 95.45% Precision (95% CI: 94.0–96.7%) while maintaining 91.19% Recall (95% CI: 89.1–93.1%), resulting in the highest F1-score and kappa. In an operational setting, this improvement is consequential because false positives directly translate into unnecessary field checks and misallocated control effort, especially in alpine wetland where background variability is high.

#### 2.2.2. Threshold Stability and the Contribution of GrMO Refinement

A practical distinction of PKI is its threshold stability. Benchmark indices require thresholds that vary by index and year (Table 1), reflecting shifts in background composition, phenology, and scene conditions that alter their value distributions. As a result, benchmark-based thresholding remains dependent on repeated threshold estimation to maintain usability in multi-year monitoring. PKI, by design, supports a single fixed decision boundary (PKI > 1) across the full time series, improving reproducibility and transferability when annual recalibration is impractical.

Comparing PKI with PKI (raw) isolates the effect of the GrMO refinement under the same rules. Although PKI (raw) already performs strongly, the refinement yields a clear improvement dominated by commission-error suppression. In the combined evaluation, Recall increases from 86.61% to 91.19.45%, accompanied by an increase in kappa from 82.73% to 87.03% (Table 1), while Precision changes only modestly (95.48% to 95.45%). This pattern indicates that GrMO primarily reduces background activation and scattered false positives without undermining the detectability of dense, spatially coherent P. kansuensis patches. The binary examples in Figure 4 are consistent with this interpretation, showing that the PKI outputs remain spatially compact and background-sparse relative to baselines, which aligns with the high-Precision, high-kappa behavior reported in Table 1.

### 2.3. Spatiotemporal Invasion Dynamics of P. kansuensis in the Bayinbuluke Alpine Wetland (2021–2025)

**Figure 5:**
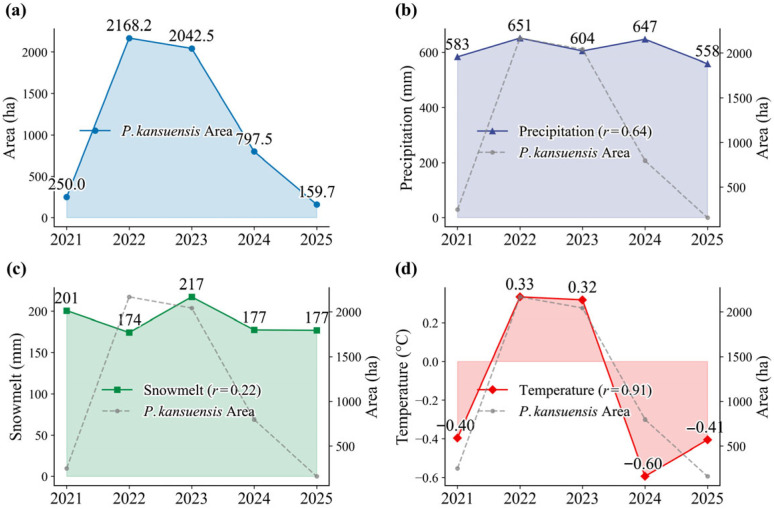
Interannual variation in the mapped invaded area of *P. kansuensis* from 2021 to 2025 (**a**), annual total precipitation (**b**), annual total snowmelt equivalent (**c**), and mean annual air temperature (**d**).

**Figure 6:**
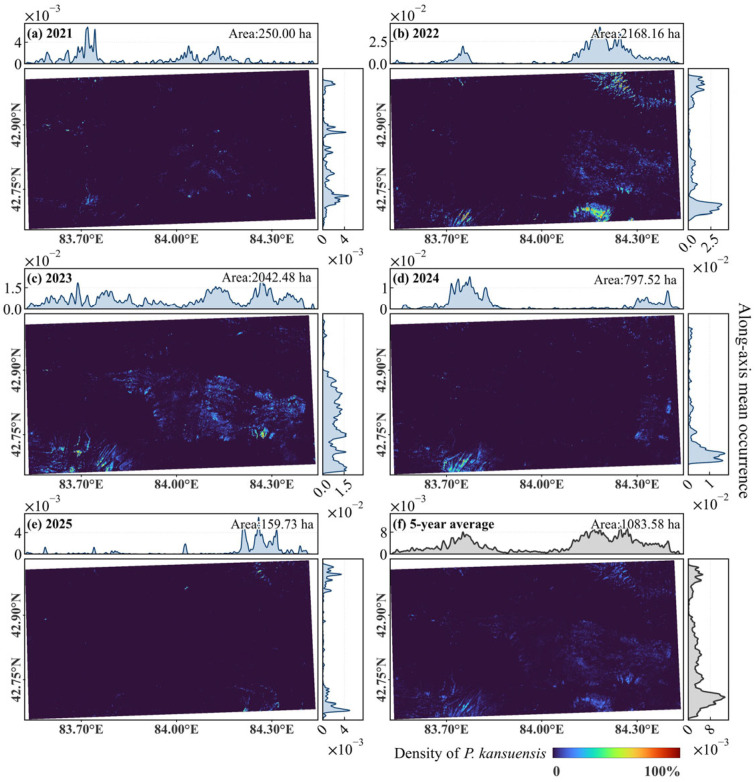
Spatial density maps of *P. kansuensis* in the Bayinbuluke alpine grassland for 2021–2025 and the 5-year mean. The curves above and right of each panel summarize the along-axis invasion probability profiles.

Based on the validated PKI-based mapping framework, the spatiotemporal dynamics of *P. kansuensis* across the Bayinbuluke alpine wetland were characterized for 2021–2025 (Figure 5 and Figure 6). The analysis integrates interannual changes in invaded extent, the persistence and reorganization of spatial concentration patterns, and the spatial structure of expansion versus contraction.

#### 2.3.1. Interannual Variability in Invaded Area

The mapped invaded area exhibits substantial interannual variability without a monotonic trend (Figure 5). Extent increased from 250.00 ha in 2021 to 2168.16 ha in 2022 and remained high in 2023 (2042.48 ha), before contracting sharply to 797.52 ha in 2024 and further to 159.73 ha in 2025. The five-year mean invaded area was 1083.58 ha. Although a linear fit suggests an overall decrease (−155.12 ha yr^−1^), the relationship is weak and non-significant (R^2^ = 0.06, p = 0.68), indicating that interannual fluctuations dominate the 2021–2025 record.

We used monthly ERA5 monthly data [29] to summarize annual total precipitation, annual total snowmelt equivalent, and mean annual air temperature over the study area, in order to briefly examine how environmental conditions may contribute to the outbreak–relaxation trajectory of *P. kansuensis* invaded area (Figure 5). A simple correlation analysis showed a strong positive relationship between invaded area and temperature (r = 0.91), suggesting that outbreaks are more likely in warmer years. This may be because higher temperatures promote earlier emergence and extend the growing season, which can accelerate population growth and spatial expansion. In addition, warming can increase snowmelt water availability, and the resulting wetter conditions may further support the growth of *P. kansuensis*.

#### 2.3.2. The Spatiotemporal Distribution and Invasion Hotspots

The annual density maps and associated probability profiles provide a comprehensive view of the intensity and spatial arrangement of *P. kansuensis* (Figure 6). In 2021, the infestation was in an incipient stage, characterized by sporadic patches primarily localized along the margins of the Bayinbuluke Grassland (Figure 6a). The marginal probability curves for this period remain relatively flat, reflecting a low-density state with minimal penetration into the landscape interior. This shifted abruptly in 2022, which functioned as the peak invaded area phase. During this year, the along-axis profiles show sharp, high-magnitude peaks, particularly along the eastern and hillside coordinates, indicating that the invasion had formed a dense and spatially continuous network (Figure 6b).

The 2023 distribution remained high in total area but exhibited a significant longitudinal shift in mass. The probability profiles for 2023 reveal a migration of the density center toward the western portions of the study area, with the peaks becoming broader and more multi-modal compared to the singular concentrations of the previous year (Figure 6c). Following this peak period, the relaxation phase in 2024 and 2025 was marked by a collapse of these density peaks. The profiles for 2025 show that the probability of occurrence declined toward baseline levels across most coordinates, leaving only residual spikes at specific hillside locations (Figure 6e).

The five-year mean density map synthesizes these annual snapshots into a representation of long-term invasion risk (Figure 6f). In summary, the region most susceptible to invasion by *P. kansuensis* are the western part of grasslands and the surrounding hillsides. The mean profiles highlight recurring core hotspots where the probability of occurrence remains consistently high despite annual fluctuations. These stable centers are likely governed by a physiographical framework of topography and hydrology that provides an ideal niche for the species. These enduring clusters serve as ecological anchors, acting as primary seed sources that facilitate rapid re-colonization whenever environmental conditions become favorable.

#### 2.3.3. Interannual Spatial Dynamics of Expansion and Contraction

**Figure 7:**
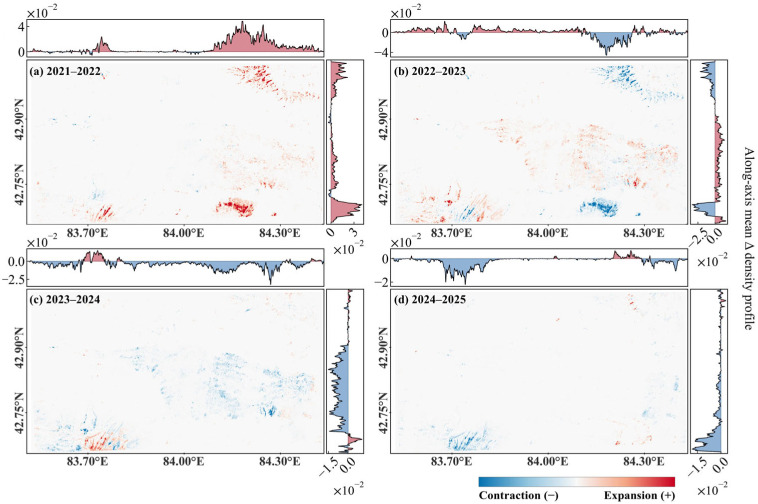
Interannual dynamics of *P. kansuensis* invasion density in the Bayinbuluke alpine grassland. The top and right curves show the mean Δ density profiles along longitude and latitude, respectively.

While the density maps describe the state of the invasion, the interannual spatial dynamics maps quantify the flux between consecutive years (Figure 7). The 2021–2022 transition was defined by overwhelming recruitment and expansion. The change profiles for this period show a massive positive surge across the entire spatial extent, particularly within previously non-invaded hillside regions (Figure 7a).

In contrast, the 2022–2023 transition was characterized by a balanced turnover or spatial rotation. The change maps reveal a mosaic of positive and negative anomalies, where expansion in the western tracts was offset by simultaneous contraction in the eastern regions (Figure 7b). The mean density change profiles during this interval oscillate around the zero-axis, statistically confirming that the invasion was undergoing a process of redistribution rather than continued net growth. This spatial rebalancing suggests that the *P. kansuensis* may have been tracking transient resource availability or responding to localized shifts in soil moisture.

A regime shift occurred during the 2023–2024 and 2024–2025 periods, which were dominated by widespread retreat and patch decay. The 2023–2024 change profiles show deep negative troughs across the landscape, indicating that the contraction was as spatially extensive as the original expansion (Figure 7c). This retreat continued into 2025, with negative changes concentrated around the margins of the remaining hotspots (Figure 7d). The few remaining positive anomalies were isolated and ephemeral, failing to form any coherent spatial structures. Collectively, these dynamics show that *P. kansuensis* is not a persistent invader.

#### 2.3.4. Implications for Monitoring and Management of Invasive Plants in Alpine Wetlands

The results from 2021 to 2025 collectively describe an outbreak-relaxation cycle. There was rapid expansion and intensified hotspots in 2022 and 2023, followed by a pronounced contraction and increasing fragmentation in 2024 and 2025. Several persistent hotspot areas remained evident in the multi-year mean (Figure 5, Figure 6 and Figure 7). This behavior underscores a significant challenge in monitoring invasions in alpine systems. Strong climatic constraints and short growing seasons can amplify year-specific environment conditions, resulting in abrupt shifts in plant expression and mapped extent. This makes risk assessments based on single-year products unstable. This indicates that management decisions based solely on the previous year’s distribution may be unreliable. In practice, while persistent hotspots can be prioritized for sustained surveillance and control, timely annual monitoring is still necessary to capture the current-year distribution and identify newly emerging patches and shifting fronts. Overall, PKI-derived products provide a multi-year depiction of *P. kansuensis* dynamics in Bayinbuluke. These products support annual status reporting, as well as the identification of persistent hotspot areas and shifting fronts relevant to long-term management.

## 3. Discussion

### 3.1. Advantages of PKI

In this study, we used in situ hyperspectral measurements to guide band selection and developed the *Pedicularis kansuensis* Index (PKI) using the blue, green, and red-edge bands of PlanetScope imagery to monitor the invasive *P. kansuensis*. The proposed PKI is simple, efficient, and robust. Compared with machine-learning and deep-learning classification methods, the PKI-based approach does not require year-by-year training samples, making it more suitable for large-area, continuous monitoring. Moreover, because of its straightforward formulation, PKI is well suited for developing automated tools for routine invasion assessment, such as user-friendly desktop software or web-based plugins. Such tools could greatly improve the efficiency of local management for *P. kansuensis.*

Another major advantage of PKI is that it is designed for PlanetScope imagery. Currently, species-level mapping of invasive plants is often carried out using UAV remote sensing [20,30,31,32]. UAV imagery provides centimeter-level spatial resolution and can capture fine structural traits of plants (e.g., leaf structure and plant size). When combined with UAV-based hyperspectral sensors, it can further improve species discrimination in both spatial and spectral domains. However, UAV surveys are difficult to apply over very large areas due to coverage limitations, such as the Bayinbuluke alpine wetlands (2841 km^2^) considered in this study. By using PlanetScope as the data source, PKI enables large-area monitoring at 3 m spatial resolution. In addition, PlanetScope’s near-daily revisit greatly improves data availability and reduces the difficulty of coordinating satellite observations with field campaigns.

### 3.2. Limitations of PKI

**Figure 8:**
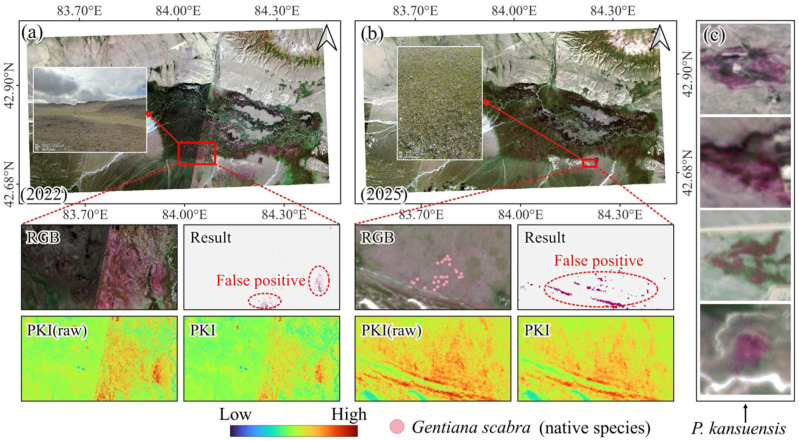
Typical sources of confusion for *P. kansuensis* mapping using PKI in the Bayinbuluke alpine grassland. (**a**,**b**) Examples of false positives caused by bare-soil and native Gentiana scabra. (**c**) The reference samples of P. *kansuensis* in PlanetScope imagery (RGB).

PKI was developed to map the invasive plant *P. kansuensis* and showed good stability and separability in the experimental evaluation. However, confusion with non-target land covers can still occur under certain conditions, leading to false positives or local errors (Figure 8).

First, false positives may be introduced by radiometric shifts caused by cross-scene normalization. For large-area monitoring with PlanetScope, images acquired on different dates are often mosaicked to achieve full spatial coverage. Because of differences in viewing geometry, illumination, atmospheric conditions, and sensor characteristics, noticeable variations in brightness and color can occur among scenes, making radiometric normalization or color balancing necessary. While such processing improves visual consistency, it can also alter the original reflectance relationships of some surfaces. Figure 8a shows a typical case in which bare soil that should have low reflectance appears unusually red in the RGB composite after normalization, which raises PKI values and produces false positives. Although PKI is formulated as a ratio to reduce multiplicative effects, additive offsets or band-inconsistent changes introduced during normalization can still distort the spectral shape. In such cases, the additive term in the numerator amplifies the error, and the ratio structure cannot fully compensate for the induced bias.

Second, a local native species, *Gentiana scabra* (see Section 4.4.1), exhibits a very similar multispectral response to *P. kansuensis* in PlanetScope bands. When G. scabra occurs at high density, even experienced experts familiar with both species and the Bayinbuluke grassland may find it difficult to distinguish them reliably using satellite imagery alone (Figure 8b,c). This reflects a common limitation of species-level mapping with multispectral data: when different species share similar visible-band coloration and comparable canopy-related responses within the available bands, index-based discrimination becomes constrained and may require higher spectral resolution data or targeted field checks.

These factors imply that PKI cannot provide error-free extraction under all circumstances. Nevertheless, PKI consistently highlights most *P. kansuensis* patches and substantially reduces the workload for large-area screening. In practice, trained local practitioners can combine terrain context, vegetation background, and patch shape to quickly flag and verify suspicious areas, allowing field surveys and control efforts to focus on high-risk locations. Overall, PKI serves as an efficient first-pass mapping tool that improves survey efficiency and supports more targeted management.

### 3.3. Invasive Habits and Recurrence Characteristics of P. kansuensis

**Figure 9:**
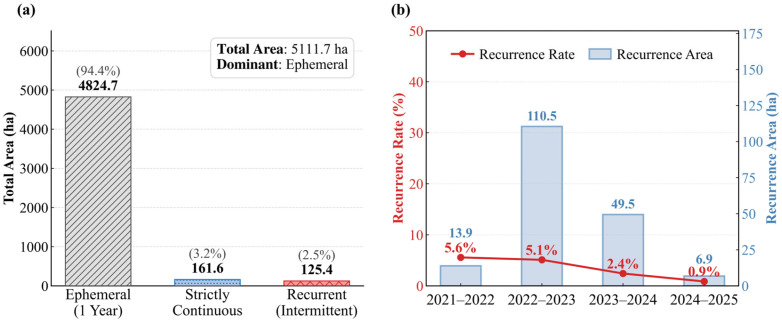
Persistence and recurrence characteristics of *P. kansuensis* occurrence derived from PKI-based annual maps (2021–2025). (**a**) Classification of the invasion footprint into three persistence types: ephemeral (present in a single year), strictly continuous (present for ≥3 consecutive years), and recurrent (intermittent reappearance). (**b**) Interannual recurrence dynamics, including recurrence rate and area.

Five consecutive years of PKI-based mapping reveal that the spatial expression of *P. kansuensis* in the Bayinbuluke alpine grassland is highly variable and largely non-persistent, implying limited predictability from year to year. Based on the 2021–2025 classification results, the invasion footprint was partitioned into three temporal persistence types (Figure 9a). The dominant component was ephemeral occurrence, accounting for 94.4% (4824.7 ha) of the total detected area (5111.7 ha), indicating that most mapped patches were present in only a single year. In contrast, only 3.2% (161.6 ha) of the area showed strict multi-year continuity (persisting for more than three years), and an additional 2.5% (125.4 ha) exhibited intermittent recurrence, i.e., re-appearance after one or more absence years. Together, these statistics suggest that *P. kansuensis* distribution is strongly shaped by interannual shifts in environmental conditions and disturbance regimes typical of high-elevation wetlands, rather than by simple spatial inertia of previously affected sites.

This dominance of one-year occurrence has direct implications for management in alpine pastoral systems. Reliance on historical “hotspots” alone is unlikely to provide reliable guidance for annual control planning, because much of the mapped area does not persist into the following growing season. In practical terms, last year’s distribution may offer only limited value for locating this year’s outbreak, particularly under the short growing season and strong climatic constraints of Bayinbuluke, where phenology and canopy expression can change rapidly. This further motivates an operational monitoring tool that can be applied consistently each season. In this context, PKI is valuable not because it enables long-term prediction, but because it supports rapid, repeatable detection of current-year occurrence, allowing control actions to be deployed in a timely manner when and where patches emerge.

Although recurrence was uncommon overall, its magnitude also declined through time (Figure 9b). Both the recurrence area and the recurrence rate decreased after 2023, suggesting a weakening tendency for re-establishment at previously affected locations. While attributing this pattern is beyond the scope of the present analysis, a plausible explanation is increasing effectiveness of local control efforts and/or changes in grazing management that reduced seed input and re-establishment success. Regardless of the driver, the low recurrence rates highlight that monitoring should emphasize early-season identification of newly emerging patches rather than focusing exclusively on historically affected zones.

### 3.4. Local Management and Control of P. kansuensis in Bayinbuluke

The Bayinbuluke grassland is a key ecological asset in arid northwestern China, supporting both pastoral production and high-value tourism. Our 2021–2025 maps provide a consistent, spatially explicit record of *P. kansuensis* dynamics during a period when local control efforts have been strengthened. Although the invaded area fluctuated markedly over the years, the most recent years show an overall decline relative to the outbreak peak. Compared with the survey-based estimate of 2.33 × 10^4^ ha reported for 2013 [25], our results, although based on a smaller study area, suggest that the extent of *P. kansuensis* has been substantially reduced. This reduction may be related to human activities (e.g., manual mowing) and/or climate factors (e.g., temperature and precipitation). However, we currently lack the quantitative records of management actions and a long time-series of *P. kansuensis* observations to identify the dominant drivers. Overall, our observations indicate a generally positive trajectory under intensified management.

Spatially, the reduction was not uniform. Contraction was most apparent within the grassland interior and parts of the higher-elevation terrain, whereas scattered patches persisted in peripheral or upslope areas. The easing of invasion pressure in the core grazing zone is consistent with the large-scale manual mowing organized locally during the flowering season, which can suppress aboveground biomass and reduce seed production. While our results do not establish causality, the coincidence between management focus and mapped contraction suggests that repeated removal has contributed to limiting the outbreak footprint.

These findings also point to a clear pathway for improving efficiency. Current operations are largely “search-based” and labor-intensive, with limited spatial guidance. With PKI and routine high-resolution imagery, control can be shifted toward monitoring-informed implementation, where annual maps delineate priority patches, guide field deployment, and support post-treatment evaluation. Finally, continued attention is warranted for upslope and peripheral occurrences, which may act as source areas and pose a downslope spread risk into more productive meadows.

## 4. Materials

### 4.1. Study Area

**Figure 10:**
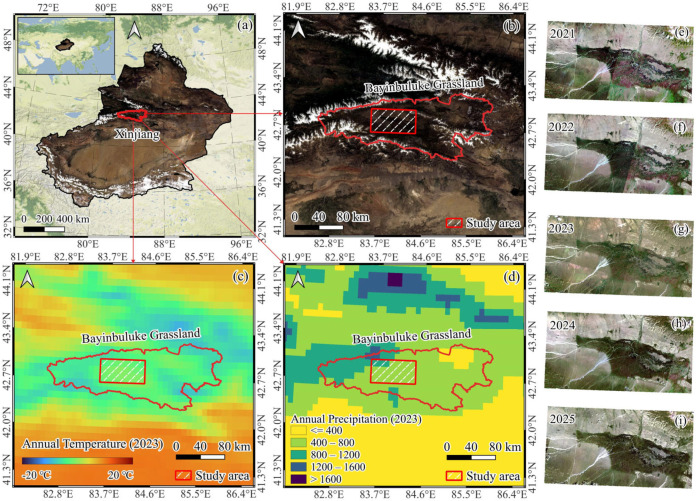
Overview of the study area (42.658–43.005° N, 83.538–84.439° E). (**a**,**b**) Geographic location of the Bayinbuluke alpine grassland and the specific study area. (**c**,**d**) Climatic characteristics of the region. (**e**–**i**) Time series of PlanetScope imagery (RGB) from 2021 to 2025.

The Bayinbuluke Grassland (42°47′ N, 84°09′ E) is located on the southern slopes of the Tianshan Mountains in northwestern China (Figure 10a,b), and represents the country’s largest alpine meadow ecosystem [18]. The region is fed by the Kaidu River and supports an extensive alpine wetland complex within an otherwise arid landscape (Figure 10d). The elevation ranges from 1500 to 2600 m, and the regional climate is classified as temperate continental arid, with strong seasonality and limited precipitation [33]. The mean annual temperature is approximately −4.7 °C (Figure 10c), reflecting a cold, high-elevation environment [34]. Bayinbuluke is widely recognized as a biodiversity hotspot in Central Asia, with records of 50 plant families, 160 genera, and more than 260 alpine plant species [35,36]. The study area (42°39′–43°00′ N, 83°32′–84°26′ E) is located in the central alpine wetlands of the Bayinbuluke Grassland and includes nearly all alpine wetlands in this region as well as a large area of surrounding pastureland, covering 2841 km^2^.

### 4.2. Pedicularis kansuensis

**Figure 11:**
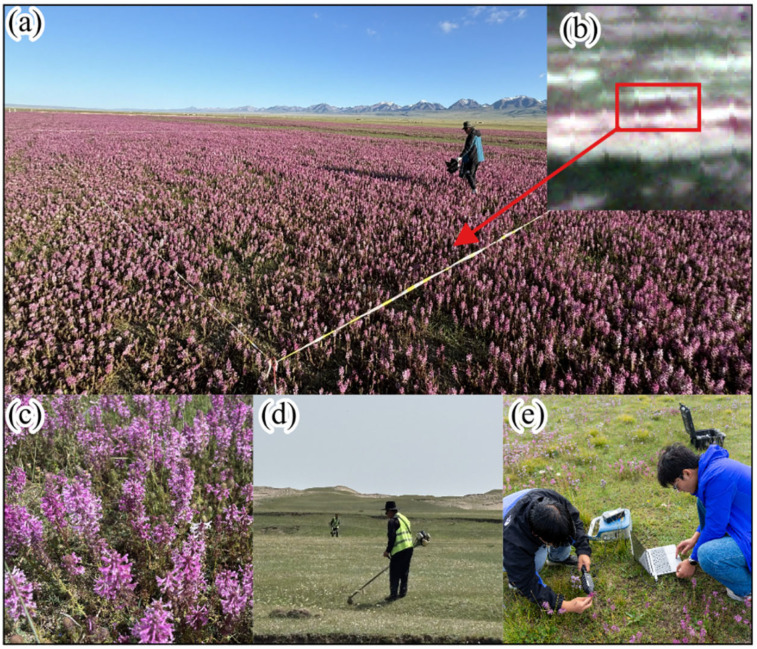
The invasive *Pedicularis kansuensis* in Bayinbuluke. (**a**) An example of an area invaded by *P. kansuensis*. (**b**) The same invaded area shown in PlanetScope imagery. (**c**) Flowers of *P. kansuensis*. (**d**) A large-scale manual cutting campaign organized by the local government to control *P. kansuensis*. (**e**) Measuring the hyperspectral reflectance spectra of *P. kansuensis* in the field.

*Pedicularis kansuensis* is an annual or biennial herb in the genus *Pedicularis* (Orobanchaceae) and is endemic to China (Figure 11). It is currently found mainly on the Qinghai–Tibet Plateau and in the Tianshan Mountains of Xinjiang. Based on our field observations (Figure 11a), *P. kansuensis* typically flowers from late July to early August, and its flowers often display a distinctive purplish-red color (Figure 11c). The species has a high seed set and strong reproductive capacity, and it commonly occurs in clustered patches. Since the early 2000s, the ecological condition of the Bayinbuluke alpine wetlands has been increasingly affected by the expansion of *P. kansuensis* [33]. Earlier studies in Xinjiang misidentified this taxon as *P. verticillata* [33]; however, recent work has confirmed that the widespread species in this region is *P. kansuensis*, which is also broadly distributed across the Qinghai–Tibet Plateau [16,37]. As a hemiparasitic herb, it has spread rapidly, degrading pasture quality, altering alpine meadow community structure, and creating socioeconomic pressures for local pastoral systems. Survey-based estimates suggest that the area affected by *P. kansuensis* has reached approximately 2.33 × 10^4^ ha, with an estimated expansion rate of about 3.30 × 10^3^ ha yr^−1^ [33]. These trends highlight the need for long-term, fine-scale monitoring of *P. kansuensis* to inform control by mapping invaded areas and expansion fronts, reducing reliance on inefficient field searches and improving management efficiency.

### 4.3. PlanetScope Imagery

To capture fine-scale invasion dynamics, multispectral imagery from the PlanetScope constellation was used [38]. The constellation consists of a large fleet of Dove microsatellites that enables high-frequency Earth observation with near-daily global revisit. This study relied on data from the next-generation SuperDove (PSB.SD) sensors, using 8-band surface reflectance products at 3 m spatial resolution [39]. The 3 m spatial resolution enables patch-level mapping of *P. kansuensis*, while the near-daily revisit increases the chance of obtaining cloud-free imagery during the short flowering period and capturing the optimal time window for detection.

The study area covers 2841 km^2^, encompassing the core distribution zone of *P. kansuensis* within the Bayinbuluke grassland. Over the five-year monitoring period (2021–2025), PlanetScope acquisitions provided a cumulative coverage of 14,204 km^2^ (Figure 10e–i) reflecting repeated observations over the area through time. All images used in this study were the 8-band Surface Reflectance product, which is already atmospherically corrected prior to release. To minimize cloud effects, we selected scenes with less than 10% cloud cover. We then mosaicked the selected scenes into one annual image using the ENVI (version 5.6) Seamless Mosaic function, applying color correction in the overlap areas.

### 4.4. Field Surveys and Validation Dataset

#### 4.4.1. Field Surveys and Ground Truth Acquisition

**Figure 12:**
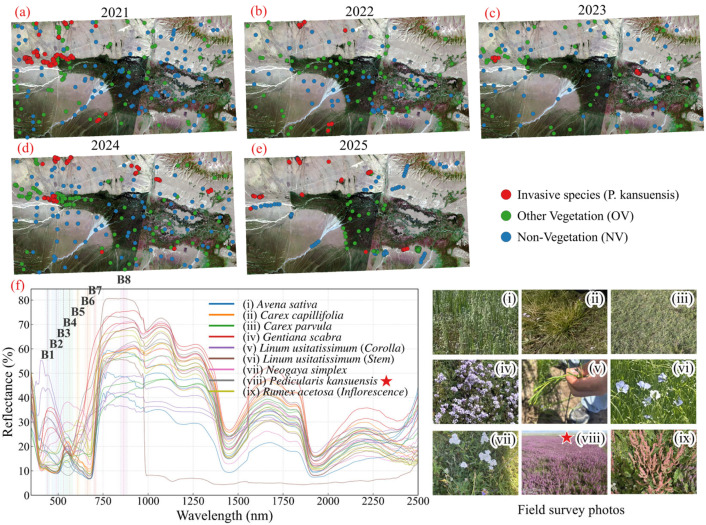
Distribution of the validation dataset and spectral characteristics of *P. kansuensis* and native species. (**a**–**e**) Spatiotemporal distribution of validation samples (2021–2025) used for accuracy assessment. (**f**) In situ hyperspectral reflectance of common vegetation types in the Bayinbuluke grassland, with field photographs (**i**–**ix**) of *P. kansuensis* (highlighted with the red star) and background vegetation.

Field surveys were conducted annually from 2023 to 2025 (Figure 12a–e), coinciding with the peak flowering stage of *P. kansuensis* (mid-July to early August). During this time, the species is most distinguishable in the field and RS imagery. Due to the vast size of the Bayinbuluke Grassland and the difficulty of accessing rugged alpine terrain, sampling was conducted along existing roads to cover areas representative of invasion severity.

Georeferenced observations of *P. kansuensis* patches were collected using handheld GPS units. At each site, we recorded the patch characteristics, including location, patch size, fractional cover, and the dominant co-occurring species. In parallel, reference locations were collected for background classes, including native species and non-vegetated surfaces. These field records were used to establish interpretation keys for PlanetScope imagery by linking the in situ observations to the corresponding satellite pixels. This supports reliable monitoring of *P. kansuensis* and provides a ground reference for dataset construction.

To examine the spectral differences between *P. kansuensis* and native species, as well as to guide the selection of sensitive bands for index design (Section 5.1), hyperspectral reflectance measurements were collected using an OFS2500 (Oceanhood, Shanghai, China) field spectroradiometer (Figure 11e and Figure 12f). This instrument covered the 350–2500 nm range at a spectral resolution of 1 nm [40]. To reduce variability related to illumination geometry and atmospheric effects, measurements were acquired under clear-sky conditions between 10:00 and 13:00 local time. The resulting in situ spectra were used to identify the wavelength regions that were most responsive to *P. kansuensis*, relative to the background vegetation. This provided a physical basis for the subsequent selection of bands and formulation of indices.

#### 4.4.2. Construction of Multi-Year *P. kansuensis* Validation Dataset

A multi-year validation dataset was compiled to validate the performance of the proposed methods. Since field observations were unavailable for 2021–2022, reference samples for these years were generated by manually interpreting PlanetScope imagery rather than using automated temporal propagation (Figure 12b–f). The interpretation criteria were based on the GPS-referenced observations acquired from 2023 to 2025. These observations provided consistent image cues for identifying *P. kansuensis* flowering clusters and distinguishing them from the native pasture.

All samples were digitized and cross-checked by the authors using consistent criteria across years, paying attention to the phenological context and scene consistency. The final dataset comprises 1682 samples, which are grouped into three classes for index validation: (1) *P. kansuensis*, which was sampled primarily from patch centers to minimize mixed-pixel effects, (2) other vegetation (OV), which represents native alpine vegetation, and (3) Non-vegetation (NV), which includes bare soil and water bodies. To ensure the fairness of validation dataset, we kept the numbers of positive samples (*P. kansuensis*) and negative samples (OV and NV) at comparable levels. The temporal distribution of the reference samples is summarized in Table 3.

## 5. Methodology

**Figure 13:**
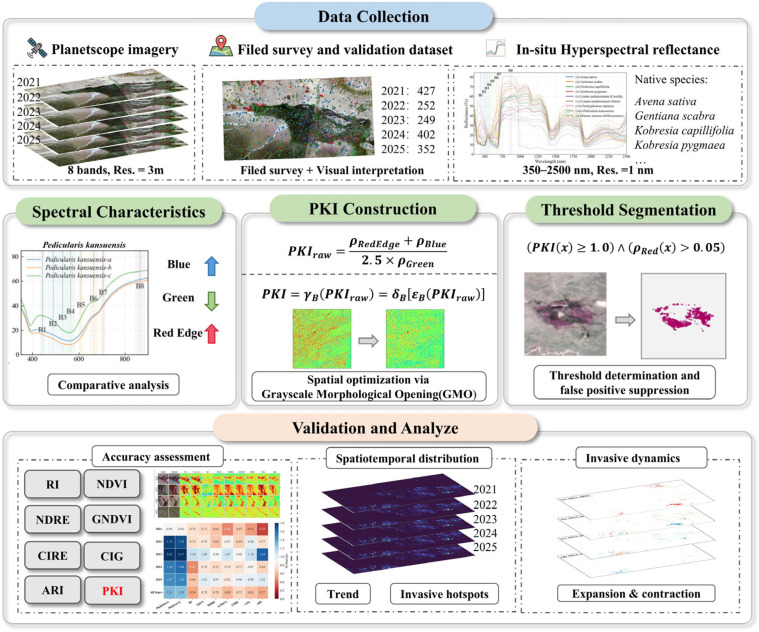
PKI-based remote sensing framework for invasive species monitoring proposed in this study.

A remote-sensing framework was developed for rapid and interpretable monitoring of *P. kansuensis* based on a species-targeted optical index (Figure 13). First, in situ hyperspectral measurements were examined to identify wavelength regions that are responsive to the flowering stage of *P. kansuensis* and compatible with the PlanetScope bands (Section 5.1). Based on these observations, a preliminary spectral index (${PKI}_{\mathrm{raw}}$) was formulated to enhance the target signal relative to native species (Section 5.2.1). To reduce high-frequency background variability in heterogeneous alpine grasslands, ${PKI}_{\mathrm{raw}}$ was further refined using a grayscale morphological opening (GrMO) to produce the final PKI (Section 5.2.2). Finally, PKI was converted into a binary map using a threshold segmentation and a low-reflectance mask (Section 5.3). For comparative evaluation, seven widely used spectral indices were implemented as benchmarks (Section 5.4).

### 5.1. Spectral Characteristics Analysis

To identify spectral features that separate *P. kansuensis* from native alpine vegetation, we analyzed in situ hyperspectral reflectance measurements collected using an OFS2500 spectroradiometer during field surveys in 2025. The analysis focused on the peak flowering period, when *P. kansuensis* shows its most distinctive canopy appearance. The observed spectral patterns were then interpreted with respect to the available PlanetScope bands to guide index development.

**Figure 14:**
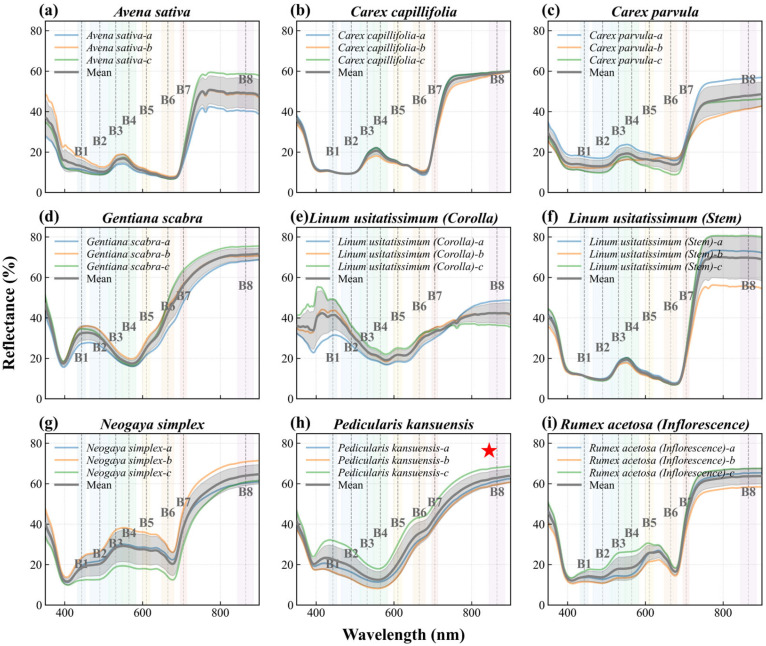
In situ spectral reflectance curves (350–900 nm) with error bars for common alpine vegetation species in the Bayinbuluke grassland, with the PlanetScope band ranges overlaid. The target species *P. kansuensis* is highlighted with a red star.

We compared the hyperspectral reflectance curve of *P. kansuensis* with those of the dominant background cover types [12], including native dominant species (*Carex capillifolia* and *Carex parvula*), cultivated species (*Avena sativa* and *Linum usitatissimum*), similarly colored species (*Gentiana scabra* and *Rumex acetosa*), and co-occurring species (*Neogaya simplex*) (Figure 14). These comparisons highlighted three consistent band-level characteristics of flowering *P. kansuensis* relative to native species: (i) higher reflectance in the Blue band, consistent with the purple-red inflorescences; (ii) a weaker Green-band peak, likely due to partial masking of green leaves by dense flowers; and (iii) a higher response across the Red and Red-Edge region, reflecting reduced Red absorption compared with chlorophyll-dominated grass canopies while retaining a strong Red-Edge signal associated with intact vegetation structure. Together, these patterns provided the basis for a species-targeted index that enhances the flowering signal of *P. kansuensis* while limiting background responses from alpine grasslands.

### 5.2. Calculation of the Pedicularis kansuensis Index (PKI)

Based on the spectral patterns summarized in Section 5.1, PKI was developed in two stages: (1) a band-combination index to enhance spectral contrast, and (2) a spatial refinement step using grayscale morphological opening to reduce noise and improve patch delineation.

#### 5.2.1. Formulation of PKI

A preliminary index, ${PKI}_{raw}$, was designed to enhance the “high Blue/high Red-Edge/low Green” pattern observed during the flowering period:

(1) $${PKI}_{raw}=\frac{\rho_{RedEdge}+\rho_{Blue}}{2.5\times \rho_{Green}}$$

where $\rho_{RedEdge}$, $\rho_{Blue}$, and $\rho_{Green}$ denote the PlanetScope surface reflectance of the Red-Edge, Blue, and Green bands, respectively. The numerator combines information from flowering-related coloration (Blue) and vegetation structural response (Red-Edge). The Green band in the denominator suppresses background grassland signals because native pastures typically exhibit stronger Green reflectance (Figure S1). A scaling factor of 2.5 was applied to normalize the threshold used to extract *P. kansuensis* (Section S3).

#### 5.2.2. Spatial Optimization via Grayscale Morphological Opening

The raw spectral indices (${PKI}_{raw}$) derived from PlanetScope imagery often exhibit speckle-like variability due to soil exposure, shadowing, sensor noise, and local heterogeneity. In contrast, *P. kansuensis* tends to occur as spatially clustered patches rather than isolated single pixels. To incorporate this spatial characteristic, grayscale morphological opening (GrMO) was applied to ${PKI}_{raw}$ image [41].

In mathematical morphology, the ${PKI}_{raw}$ image is treated as a topographic surface where pixel values correspond to elevations. The GrMO operator γ applies grayscale erosion followed by grayscale dilation over a flat structuring element B, which functions as a sliding local window defining the neighborhood for the operation:

(2) $$PKI=\gamma_{B}(PKI_{raw})=\delta_{B}\left[\varepsilon_{B}(PKI_{raw})\right]$$

where $\varepsilon_{B}$ represents the erosion operator and $\delta_{B}$ represents the dilation operator. These operations are mathematically defined as follows:

(1) Grayscale Erosion ($\varepsilon_{B}$): This operation first acts on the input $PKI_{raw}$ to suppress bright features smaller than the structuring element. For a pixel at spatial location x, the value is replaced by the minimum value of $PKI_{raw}$ found within the neighborhood defined by B:

(3) $$\left[\varepsilon_{B}(PKI_{raw})](x)=\underset{z\in B}{min}PKI_{raw}(x+z\right)$$

where z represents the spatial offset within the window B. Ecologically, this step effectively eliminates isolated noise spikes and spatially incoherent high-intensity artifacts that lack the geometric continuity of authentic vegetation communities.

(2) Grayscale Dilation ($\delta_{B}$): Following erosion, the dilation operator acts on the eroded image (denoted here as $PKI_{raw}$) to reconstruct the boundaries of the remaining features. It replaces the pixel value with the maximum value in the same neighborhood:

(4) $$\left[\delta_{B}(PKI_{ero})](x)=\underset{z\in B}{max}PKI_{ero}(x+z\right)$$

This step restores the approximate size and shape of the valid *P. kansuensis* patches that were preserved during the erosion phase, preventing the underestimation of the target area coverage.

In this study, the structuring element B was defined as a flat, square 3 × 3 pixel sliding window (Section S4). Consequently, the final PKI functions as a “spatial sieve,” filtering out high-frequency spectral noise from $PKI_{raw}$ while strictly preserving the geometric integrity of the target communities.

### 5.3. Threshold Determination and False Positive Suppression

A key design goal of PKI is operational simplicity. Rather than relying on complex classifiers or year-specific tuning, we deliberately adopt an extremely simple thresholding scheme to test whether the index itself provides sufficient separability for practical monitoring. Specifically, PKI values were converted into a binary *P. kansuensis* map using a single fixed threshold derived from ground-referenced samples, with T = 1.0. Under this rule, pixels with PKI ≥ 1.0 are labeled as *P. kansuensis*, reflecting that the flowering-enhanced signal emphasized by PKI exceeds the weighted background term by construction.

As with many ratio-based indices, artificially high values can occur over very dark non-vegetated surfaces when the denominator is close to zero. In the Bayinbuluke alpine wetland environment, deep shadows, water, and dark moist soil may therefore produce isolated false positives. To suppress these cases while keeping the procedure simple and interpretable, we added a single reflectance validity check based on the Red band. Because flowering *P. kansuensis* maintains a measurable red reflectance (Section 5.1), pixels with $\rho_{Red}\leq 0.05$ were excluded as non-vegetation artifacts. The final rule remains a straightforward logical conjunction:

(5) $$\mathrm{Class}(x)=\left\{\begin{array}{l} P.\;kansuensis,\mathrm{if}(PKI(x)\geq 1.0)\wedge (\rho_{Red}(x)>0.05)\\ \mathrm{Background},\;\mathrm{otherwise} \end{array}\right.$$

This lightweight segmentation provides a transparent baseline for evaluating PKI, where mapping performance is primarily governed by index separability under a fixed decision rule, rather than by classifier complexity or year-specific tuning.

### 5.4. Comparative Analysis

To benchmark PKI, we compared it with several widely used spectral indices that capture complementary vegetation information in PlanetScope imagery. All indices were evaluated with standard accuracy metrics to provide a consistent basis for comparison.

#### 5.4.1. Backbone Indices

To evaluate the effectiveness of PKI against commonly used approaches, we selected a set of established vegetation and pigment-related indices as benchmarks (Table 4). The selected indices span major spectral sensitivity types available in PlanetScope imagery, including greenness-based vigor measures, red-edge chlorophyll proxies, and visible-band indices related to coloration.

RI [42] was included as a simple visible-band ratio that responds to leaf and flower color changes and is therefore informative for the reddish-purplish flowers of *P. kansuensis*. NDVI [43] and GNDVI [44] were used as standard greenness-based indices derived from NIR band differences with the Red or Green bands, providing baselines for vegetation vigor and biomass. To account for chlorophyll-related variation in dense alpine grasslands, NDRE [45] was included as a Red-Edge normalized difference index that is generally less prone to saturation than NDVI, and CIRE was included as a Red-Edge chlorophyll index. CIG [46] was further included as a Green-band chlorophyll index comparable to CIRE [47]. Finally, ARI [48] was included as an anthocyanin-related index using the Green and Red-Edge bands, reflecting pigment responses associated with red, blue, and purple coloration.

All benchmark indices were computed from PlanetScope surface reflectance (ρ) using standard definitions, and their formulas and intended sensitivities are summarized in Table 4.

#### 5.4.2. Accuracy Metrics

Classification performance was evaluated using Accuracy, F1-score, Cohen’s kappa, Precision, and Recall. These metrics are widely used in remote sensing classification because they jointly describe error balance, overall correctness, and agreement beyond chance, providing a robust basis for comparing threshold-segmentation maps derived from different indices [49].

#### 5.4.3. M-Statistic

To quantify how well each index separates *P. kansuensis* from non-target classes at the sample level, the M-statistic (M) was used as a distribution-based separability metric [50]. Unlike accuracy measures that depend on a chosen threshold, the M-statistic evaluates the inherent distance between two class distributions and is therefore well suited for comparing indices with different value ranges. For a given index, M was computed between the *P. kansuensis* samples and each background group (OV and NV) based on their sample histograms, following the standard definition:

(6) $$M=\frac{\left|\mu_{1}-\mu_{2}\right|}{\sigma_{1}+\sigma_{2}}$$

where $\mu_{1}$ and $\mu_{2}$ denote the mean index values of the two classes being compared, and $\sigma_{1}$ and $\sigma_{2}$ denote their corresponding standard deviations. Larger M values indicate greater separability, while smaller values imply stronger overlap between class distributions. Consistent with common practice, (M > 1.0) was interpreted as good separability, whereas (M < 1.0) indicates substantial overlap and limited discrimination potential. In this study, M was calculated for each year and for the full combined dataset to enable consistent inter-index comparisons under varying background composition and acquisition conditions.

## 6. Conclusions

This study presents a practical, species-level, remote sensing approach to monitoring the invasive species *Pedicularis kansuensis* in alpine wetlands of the Bayinbuluke grassland using PlanetScope imagery. We designed the *Pedicularis kansuensis* Index (PKI) by aligning in situ hyperspectral observations with the PlanetScope band configuration. The PKI emphasizes the characteristic flowering-season response of *P. kansuensis* while reducing the influence of background vegetation and non-vegetated dark surfaces through a spatial refinement step using grayscale morphological opening (GrMO).

Based on a comprehensive evaluation using the 2021–2025 validation dataset, PKI consistently outperformed seven commonly used indices in discriminating *P. kansuensis* under heterogeneous alpine backgrounds. M-statistic results indicate that PKI provides the highest sample-level separability for *P. kansuensis* (mean M = 1.36). Comparisons of classification accuracy demonstrate PKI’s superior performance, achieving a five-year mean overall accuracy of 93.52% (95% CI: 92.3–94.7%) and a mean F1 score of 93.28% (95% CI: 92.0–94.5%). Notably, PKI relies on a single, physically interpretable decision rule (PKI > 1) and requires no year-specific re-tuning. Finally, a comparison between PKI (raw) and PKI confirms the effect of GrMO. GrMO primarily improves performance by suppressing non-target responses while retaining dense, spatially coherent *P. kansuensis* signals.

Time-series mapping using PKI reveals the outbreak-relaxation patten of the invasion from 2021 to 2025. The area affected by the invasion exhibited a marked increase, rising from 250 hectares (ha) in 2021 to 2168 ha in 2022, and remained high in 2023 at 2042 ha. This was followed by substantial contractions in 2024 (797 ha) and 2025 (159 ha). This behavior suggests that the invasion status of *P. kansuensis* can change abruptly from year to year. Spatial analysis identified 161.6 hectares of stable invasion hotspots, thereby highlighting key targets for long-term containment. Concurrently, 94.4% of the invaded area was transient, with a duration of only one year (4824.7 ha).

## Figures and Tables

**Table 1:** Threshold segmentation results of the proposed indices (PKI and PKI (raw), highlighted in red) and seven benchmark indices (RI, NDVI, NDRE, GNDVI, CIRE, CIG, and ARI) for *P. kansuensis* detection across 2021–2025 and the entire dataset. Green indicates better performance, while red indicates poorer performance.

Year	Metric	PKI	PKI(raw)	RI	NDVI	NDRE	GNDVI	CIRE	CIG	ARI
2021	Accuracy	87.82%	77.99%	67.21%	65.11%	62.53%	66.28%	67.92%	65.81%	66.04%
2021	F1-Score	87.06%	74.03%	75.69%	74.79%	73.33%	75.26%	76.01%	73.93%	72.80%
2021	Kappa	75.77%	56.49%	32.86%	28.37%	22.96%	30.88%	34.36%	30.17%	30.97%
2021	Precision	96.69%	95.04%	61.41%	59.73%	58.05%	60.66%	62.00%	61.06%	62.18%
2021	Recall	79.19%	60.63%	98.64%	100.00%	99.55%	99.10%	98.19%	93.67%	87.78%
2021	Lower_Limit	1.0000	1.0000	0.7800	0.4657	0.2880	0.5562	0.8088	2.5063	9.9972
2021	Upper_Limit	inf	inf	1.4932	0.8711	0.6138	0.8596	2.6572	7.9741	17.9092
2022	Accuracy	95.63%	94.44%	72.22%	74.60%	67.86%	69.84%	67.86%	69.84%	69.84%
2022	F1-Score	94.69%	93.20%	73.68%	75.38%	70.76%	72.06%	70.76%	72.06%	72.06%
2022	Kappa	91.00%	88.52%	48.28%	52.24%	41.21%	44.39%	41.21%	44.39%	44.39%
2022	Precision	89.91%	88.89%	58.33%	60.49%	54.75%	56.32%	54.75%	56.32%	56.32%
2022	Recall	100.00%	97.96%	100.00%	100.00%	100.00%	100.00%	100.00%	100.00%	100.00%
2022	Lower_Limit	1.0000	1.0000	0.9882	0.5610	0.3551	0.6489	1.1015	3.6959	12.6576
2022	Upper_Limit	inf	inf	1.6490	0.8011	0.5624	0.8336	2.5702	10.0183	24.1921
2023	Accuracy	98.80%	98.39%	65.46%	77.91%	75.90%	78.71%	77.51%	78.71%	82.73%
2023	F1-Score	98.80%	98.41%	74.25%	81.61%	79.59%	81.40%	80.00%	81.00%	84.91%
2023	Kappa	97.59%	96.79%	31.11%	55.90%	51.88%	57.48%	55.07%	57.47%	65.50%
2023	Precision	97.64%	96.88%	59.05%	69.71%	68.82%	72.05%	71.79%	72.90%	75.16%
2023	Recall	100.00%	100.00%	100.00%	98.39%	94.35%	93.55%	90.32%	91.13%	97.58%
2023	Lower_Limit	1.0000	1.0000	0.9492	0.5116	0.3205	0.6505	0.9435	3.7218	11.2442
2023	Upper_Limit	inf	inf	1.9502	0.7760	0.5171	0.7920	1.9140	6.9776	20.8759
2024	Accuracy	96.27%	96.52%	58.21%	72.89%	70.40%	75.37%	70.15%	72.89%	72.64%
2024	F1-Score	96.33%	96.59%	70.53%	79.32%	77.84%	80.78%	77.36%	79.16%	78.93%
2024	Kappa	92.54%	93.03%	13.68%	44.48%	39.27%	49.70%	38.85%	44.53%	44.03%
2024	Precision	98.50%	98.51%	55.68%	65.72%	63.72%	67.97%	63.86%	65.92%	65.81%
2024	Recall	94.26%	94.74%	96.17%	100.00%	100.00%	99.52%	98.09%	99.04%	98.56%
2024	Lower_Limit	1.0000	1.0000	0.7963	0.4759	0.2818	0.6031	0.7848	2.3443	7.9657
2024	Upper_Limit	inf	inf	1.4292	0.8738	0.6157	0.8635	2.9182	11.1221	23.8457
2025	Accuracy	92.05%	94.60%	63.92%	79.83%	77.84%	78.98%	78.41%	82.67%	86.65%
2025	F1-Score	92.05%	94.59%	73.60%	83.14%	81.34%	82.21%	81.46%	84.56%	87.98%
2025	Kappa	84.09%	89.21%	27.54%	59.57%	55.59%	57.87%	56.74%	65.29%	73.26%
2025	Precision	92.57%	95.40%	58.22%	71.72%	70.54%	71.55%	71.67%	76.61%	80.37%
2025	Recall	91.53%	93.79%	100.00%	98.87%	96.05%	96.61%	94.35%	94.35%	97.18%
2025	Lower_Limit	1.0000	1.0000	0.7033	0.6011	0.3695	0.6644	1.1719	3.7324	13.1888
2025	Upper_Limit	inf	inf	1.4442	0.8612	0.5557	0.8205	2.3409	7.6972	21.7602
All Years	Accuracy	93.52%	91.38%	57.31%	66.94%	65.46%	68.79%	68.43%	65.52%	67.30%
All Years	F1-Score	93.28%	90.83%	69.24%	74.89%	74.05%	75.91%	75.45%	73.32%	74.79%
All Years	Kappa	87.03%	82.73%	15.58%	34.49%	31.58%	38.11%	37.38%	31.62%	35.16%
All Years	Precision	95.45%	95.48%	53.69%	59.86%	58.79%	61.26%	61.17%	59.26%	60.31%
All Years	Recall	91.19%	86.61%	97.47%	100.00%	100.00%	99.76%	98.43%	96.14%	98.43%
All Years	Lower_Limit	1.0000	1.0000	0.7136	0.4657	0.2818	0.5833	0.7848	2.3443	7.9657
All Years	Upper_Limit	inf	inf	1.6456	0.8975	0.6288	0.8672	2.8116	9.4619	24.3724

**Table 2:** The 95% confidence interval of accuracy metrics of proposed indices (PKI and PKI (raw), highlighted in red) and seven benchmark indices (RI, NDVI, NDRE, GNDVI, CIRE, CIG, and ARI) for *P. kansuensis* detection across 2021–2025 and the entire dataset. Green indicates better performance, while red indicates poorer performance.

Year	Metric	PKI	PKI(raw)	RI	NDVI	NDRE	GNDVI	CIRE	CIG	ARI
2021	Accuracy	[84.3%, 90.9%]	[73.8%, 82.0%]	[61.6%, 73.3%]	[61.7%, 74.2%]	[59.6%, 73.7%]	[62.1%, 73.1%]	[64.6%, 75.3%]	[60.9%, 73.8%]	[61.6%, 74.5%]
2021	F1-Score	[83.3%, 90.3%]	[68.6%, 79.0%]	[71.4%, 80.0%]	[71.6%, 80.9%]	[70.6%, 80.1%]	[71.8%, 80.0%]	[72.9%, 81.3%]	[69.9%, 79.0%]	[69.2%, 79.0%]
2021	Kappa	[69.1%, 81.6%]	[48.6%, 64.4%]	[23.1%, 44.7%]	[23.4%, 47.5%]	[19.1%, 45.0%]	[24.2%, 44.2%]	[29.0%, 48.8%]	[22.4%, 45.5%]	[24.4%, 48.1%]
2021	Precision	[93.8%, 99.0%]	[91.2%, 98.2%]	[55.8%, 67.6%]	[55.8%, 68.1%]	[54.6%, 67.0%]	[56.4%, 67.3%]	[58.2%, 69.8%]	[55.8%, 69.1%]	[57.1%, 71.1%]
2021	Recall	[73.5%, 84.3%]	[54.0%, 67.3%]	[95.6%, 100%]	[98.7%, 100%]	[97.8%, 100%]	[94.3%, 100%]	[94.5%, 99.8%]	[89.5%, 96.3%]	[83.7%, 93.3%]
2021	Lower_Limit	[1.00, 1.00]	[1.00, 1.00]	[0.75, 0.87]	[0.47, 0.53]	[0.29, 0.33]	[0.56, 0.60]	[0.81, 0.96]	[2.51, 3.00]	[10.00, 11.80]
2021	Upper_Limit	[1.00, Inf]	[1.00, Inf]	[1.39, 1.49]	[0.83, 0.87]	[0.56, 0.63]	[0.80, 0.87]	[2.32, 2.76]	[6.70, 8.99]	[16.71, 20.17]
2022	Accuracy	[92.5%, 98.0%]	[91.3%, 97.2%]	[67.9%, 80.6%]	[69.4%, 80.0%]	[63.1%, 75.0%]	[64.9%, 77.8%]	[63.1%, 75.0%]	[64.7%, 76.0%]	[63.9%, 77.4%]
2022	F1-Score	[91.2%, 97.6%]	[88.9%, 96.6%]	[68.9%, 81.2%]	[69.5%, 81.0%]	[65.2%, 77.2%]	[66.8%, 78.5%]	[65.2%, 77.2%]	[66.4%, 78.1%]	[65.7%, 78.7%]
2022	Kappa	[84.6%, 95.9%]	[82.2%, 94.2%]	[41.6%, 62.5%]	[44.0%, 61.4%]	[33.7%, 52.5%]	[36.9%, 57.4%]	[33.7%, 52.5%]	[36.7%, 54.4%]	[35.4%, 56.9%]
2022	Precision	[83.8%, 95.4%]	[82.4%, 94.8%]	[52.5%, 68.3%]	[53.3%, 68.1%]	[48.4%, 62.9%]	[50.1%, 65.6%]	[48.4%, 62.9%]	[49.8%, 64.2%]	[48.9%, 65.3%]
2022	Recall	[100%, 100%]	[94.9%, 100%]	[100%, 100%]	[100%, 100%]	[100%, 100%]	[96.1%, 100%]	[100%, 100%]	[96.3%, 100%]	[91.3%, 100%]
2022	Lower_Limit	[1.00, 1.00]	[1.00, 1.00]	[0.99, 1.06]	[0.56, 0.59]	[0.36, 0.38]	[0.65, 0.69]	[1.10, 1.25]	[3.70, 4.05]	[12.66, 14.04]
2022	Upper_Limit	[1.00, Inf]	[1.00, Inf]	[1.53, 1.65]	[0.79, 0.80]	[0.56, 0.56]	[0.83, 0.83]	[2.52, 2.57]	[9.47, 10.02]	[22.95, 24.19]
2023	Accuracy	[97.2%, 100%]	[96.8%, 99.6%]	[61.0%, 81.9%]	[72.7%, 83.1%]	[72.3%, 84.7%]	[74.7%, 85.1%]	[72.5%, 84.7%]	[73.9%, 84.3%]	[79.5%, 92.8%]
2023	F1-Score	[97.2%, 100%]	[96.8%, 99.6%]	[69.8%, 84.6%]	[76.9%, 86.2%]	[76.0%, 85.5%]	[77.5%, 87.0%]	[75.6%, 85.1%]	[76.2%, 86.0%]	[81.4%, 93.3%]
2023	Kappa	[94.4%, 100%]	[93.6%, 99.2%]	[23.9%, 64.4%]	[46.2%, 65.7%]	[45.0%, 69.5%]	[49.8%, 70.1%]	[45.1%, 69.5%]	[47.6%, 68.1%]	[58.9%, 85.5%]
2023	Precision	[94.5%, 100%]	[93.7%, 99.3%]	[53.7%, 73.9%]	[63.3%, 77.5%]	[63.4%, 85.7%]	[67.3%, 79.7%]	[63.7%, 86.3%]	[67.3%, 80.3%]	[69.6%, 89.1%]
2023	Recall	[100%, 100%]	[100%, 100%]	[96.7%, 100%]	[91.9%, 100%]	[81.4%, 100%]	[88.0%, 100%]	[80.2%, 100%]	[84.9%, 95.7%]	[94.1%, 100%]
2023	Lower_Limit	[1.00, 1.00]	[1.00, 1.00]	[0.95, 1.11]	[0.51, 0.53]	[0.32, 0.33]	[0.65, 0.66]	[0.94, 0.96]	[3.72, 3.91]	[11.24, 13.03]
2023	Upper_Limit	[1.00, Inf]	[1.00, Inf]	[1.74, 1.95]	[0.74, 0.80]	[0.42, 0.55]	[0.76, 0.82]	[1.42, 2.49]	[5.99, 8.21]	[18.88, 21.12]
2024	Accuracy	[94.3%, 98.0%]	[94.8%, 98.0%]	[53.0%, 64.7%]	[69.0%, 78.0%]	[66.4%, 75.6%]	[71.1%, 79.4%]	[65.7%, 75.4%]	[68.9%, 78.6%]	[68.9%, 80.1%]
2024	F1-Score	[94.1%, 98.0%]	[94.6%, 98.2%]	[66.0%, 75.6%]	[75.7%, 83.5%]	[74.1%, 82.0%]	[76.9%, 84.4%]	[73.6%, 82.0%]	[75.7%, 83.5%]	[75.6%, 84.3%]
2024	Kappa	[88.6%, 96.0%]	[89.4%, 96.0%]	[7.3%, 24.3%]	[38.2%, 53.7%]	[32.7%, 48.0%]	[42.2%, 56.7%]	[31.3%, 47.3%]	[37.3%, 55.1%]	[37.8%, 58.8%]
2024	Precision	[96.5%, 100%]	[96.5%, 100%]	[50.5%, 62.0%]	[60.9%, 71.9%]	[58.9%, 69.5%]	[62.7%, 73.3%]	[59.1%, 69.9%]	[61.1%, 72.2%]	[61.5%, 73.6%]
2024	Recall	[91.1%, 97.3%]	[91.8%, 97.6%]	[91.0%, 99.5%]	[96.9%, 100%]	[98.2%, 100%]	[98.5%, 100%]	[95.9%, 100%]	[97.0%, 100%]	[96.7%, 100%]
2024	Lower_Limit	[1.00, 1.00]	[1.00, 1.00]	[0.76, 0.88]	[0.48, 0.55]	[0.28, 0.31]	[0.60, 0.61]	[0.78, 0.85]	[2.34, 3.10]	[7.97, 10.09]
2024	Upper_Limit	[1.00, Inf]	[1.00, Inf]	[1.35, 1.49]	[0.86, 0.87]	[0.60, 0.62]	[0.84, 0.86]	[2.75, 3.20]	[9.82, 12.65]	[22.52, 26.38]
2025	Accuracy	[89.5%, 94.6%]	[92.0%, 97.0%]	[59.7%, 74.1%]	[75.3%, 86.8%]	[74.4%, 83.2%]	[74.7%, 85.2%]	[74.7%, 83.8%]	[78.7%, 87.1%]	[83.2%, 89.8%]
2025	F1-Score	[89.0%, 94.9%]	[91.8%, 96.9%]	[69.5%, 80.5%]	[79.4%, 88.3%]	[77.3%, 86.2%]	[78.7%, 87.2%]	[77.9%, 86.1%]	[80.9%, 88.6%]	[84.5%, 91.1%]
2025	Kappa	[78.9%, 89.2%]	[84.1%, 94.0%]	[21.9%, 46.9%]	[50.1%, 73.1%]	[48.8%, 66.1%]	[49.7%, 69.7%]	[49.7%, 66.9%]	[57.4%, 74.0%]	[66.6%, 79.6%]
2025	Precision	[88.5%, 96.2%]	[91.8%, 98.3%]	[53.4%, 67.6%]	[66.5%, 80.2%]	[65.7%, 77.7%]	[66.2%, 79.6%]	[66.5%, 78.9%]	[71.0%, 82.9%]	[75.9%, 85.7%]
2025	Recall	[87.1%, 95.6%]	[90.2%, 97.0%]	[98.2%, 100%]	[95.8%, 100%]	[92.2%, 98.9%]	[92.0%, 99.4%]	[90.6%, 97.6%]	[91.1%, 97.6%]	[93.2%, 98.9%]
2025	Lower_Limit	[1.00, 1.00]	[1.00, 1.00]	[0.70, 0.84]	[0.60, 0.61]	[0.37, 0.38]	[0.65, 0.68]	[1.17, 1.24]	[3.73, 3.98]	[13.19, 13.43]
2025	Upper_Limit	[1.00, Inf]	[1.00, Inf]	[1.41, 1.44]	[0.82, 0.89]	[0.53, 0.57]	[0.79, 0.83]	[2.21, 2.59]	[7.28, 8.55]	[19.98, 21.76]
All Years	Accuracy	[92.3%, 94.7%]	[89.9%, 92.7%]	[54.9%, 62.4%]	[65.1%, 70.0%]	[63.8%, 68.8%]	[66.9%, 71.9%]	[66.4%, 71.0%]	[63.6%, 69.2%]	[65.5%, 72.6%]
All Years	F1-Score	[92.0%, 94.5%]	[88.9%, 92.2%]	[67.3%, 72.2%]	[73.0%, 77.2%]	[72.4%, 76.3%]	[74.1%, 78.3%]	[73.5%, 77.5%]	[71.3%, 76.1%]	[73.0%, 78.3%]
All Years	Kappa	[84.6%, 89.4%]	[79.7%, 85.3%]	[12.4%, 24.9%]	[32.1%, 39.9%]	[29.3%, 37.3%]	[35.4%, 44.2%]	[34.2%, 42.0%]	[28.7%, 38.6%]	[32.1%, 45.7%]
All Years	Precision	[94.0%, 96.7%]	[94.0%, 96.8%]	[51.5%, 57.5%]	[57.5%, 62.9%]	[56.8%, 61.7%]	[58.9%, 64.4%]	[58.7%, 63.9%]	[56.9%, 62.6%]	[58.2%, 65.1%]
All Years	Recall	[89.1%, 93.1%]	[83.9%, 88.8%]	[95.4%, 98.9%]	[100%, 100%]	[99.6%, 100%]	[99.3%, 100%]	[97.3%, 99.2%]	[94.2%, 98.4%]	[97.0%, 99.1%]
All Years	Lower_Limit	[1.00, 1.00]	[1.00, 1.00]	[0.69, 0.78]	[0.47, 0.49]	[0.28, 0.30]	[0.58, 0.60]	[0.78, 0.85]	[2.34, 2.99]	[7.97, 10.04]
All Years	Upper_Limit	[1.00, Inf]	[1.00, Inf]	[1.57, 1.68]	[0.87, 0.90]	[0.61, 0.63]	[0.86, 0.87]	[2.67, 2.96]	[8.96, 9.99]	[23.30, 24.82]

**Table 3:** Number of sample points across the study period (2021–2025).

Year	*P. kansuensis*	Other Vegetation	Non- Vegetation	Total
2021	221	98	108	427
2022	98	92	62	252
2023	124	67	58	249
2024	209	98	95	402
2025	177	91	84	352
Total	829	446	407	1682

**Table 4:** Definitions of the proposed PKI and the benchmark spectral indices used in this study.

Index	Full Name	Formula	Description
RI	Red Index	$\frac{\rho_{Red}}{\rho_{Green}}$	Simple ratio sensitive to leaf/flower color changes, captures the “redness” of inflorescences.
NDVI	Normalized Difference Vegetation Index	$\frac{\rho_{NIR}-\rho_{Red}}{\rho_{NIR}+\rho_{Red}}$	Standard proxy for vegetation greenness and biomass.
NDRE	Normalized Difference Red Edge	$\frac{\rho_{NIR}-\rho_{RedEdge}}{\rho_{NIR}+\rho_{RedEdge}}$	Sensitive to chlorophyll content, less prone to saturation than NDVI in dense grass.
GNDVI	Green NDVI	$\frac{\rho_{NIR}-\rho_{Green}}{\rho_{NIR}+\rho_{Green}}$	Indicates chlorophyll concentration, uses Green channel instead of Red.
CIRE	Chlorophyll Index Red-Edge	$\frac{\rho_{NIR}}{\rho_{RedEdge}}-1$	Estimator of canopy chlorophyll content using the red-edge band.
CIG	Chlorophyll Index Green	$\frac{\rho_{NIR}}{\rho_{Green}}-1$	Estimator of chlorophyll using the green band, comparable to CIRE.
ARI	Anthocyanin Reflectance Index	$\frac{1}{\rho_{Green}}-\frac{1}{\rho_{RedEdge}}$	Estimator of anthocyanins using the green and red-edge bands, associated with red, blue, and purple pigmentation.
PKI (raw)	*P. kansuensis* Index (raw)	$\frac{\rho_{RedEdge}+\rho_{Blue}}{2.5\times \rho_{Green}}$	Spectral-contrast index tailored to enhance flowering-season *P. kansuensis* signals while suppressing green-dominated native vegetation.
PKI (ours)	*P. kansuensis* Index (PKI)	$\delta_{B}\left[\varepsilon_{B}(PKI_{raw})\right]$	Spatially refined PKI using grayscale morphological opening to reduce background fluctuations and improve patch delineation.

## Data Availability

PlanetScope data used in this study are available from Planet Labs (https://www.planet.com/).

## Supplementary Materials

The following supporting information can be downloaded at: https://www.mdpi.com/article/10.3390/plants15050806/s1, Figure S1: The Jeffries–Matusita (JM) distance of P. kansuensis and background pixels at each PlanetScope band; Figure S2: Unscaled PKI distribution (pixels of P. kansuensis and background vegetation); Figure S3: The performance of GrMO with different kernel size.
