# Supplementary material for: Monitoring the Spatiotemporal Dynamics of Invasive Pedicularis kansuensis in Bayinbuluke Alpine Wetlands: A Novel Spectral Index Framework Using PlanetScope Time Series (2021–2025)
Source: Plants (Basel). 2026 Mar 6;15(5):806. doi: 10.3390/plants15050806 (PMC12987086; doi:10.3390/plants15050806)
Supplement: Supplementary file 1 [file plants-15-00806-s001.zip › plants-4133439-supplementary.docx]

**Monitoring the spatiotemporal dynamics of the invasive plant *Pedicularis kansuensis* in alpine wetlands: A novel spectral index approach using PlanetScope time series (2021–2025)**

# **Supplementary Materials**

# **S1. Selection of the Scaling Factor (2.5)**

In this study, we added a scaling factor of 2.5 to the denominator of the original spectral index of PKI raw. In practice, the scaling factor is an empirical coefficient used to normalize the segmentation threshold, making the index easier to interpret and apply. As shown in **Fig. S2**, in the unscaled PKI distribution for all samples (bottom-right panel), the curves for P. kansuensis and the background vegetation intersect at around 2.5. If the scaling factor is set higher than this value, many target samples would be missed; if it is set lower, more background errors would be introduced. Therefore, the preferred scaling factor should be close to 2.5.


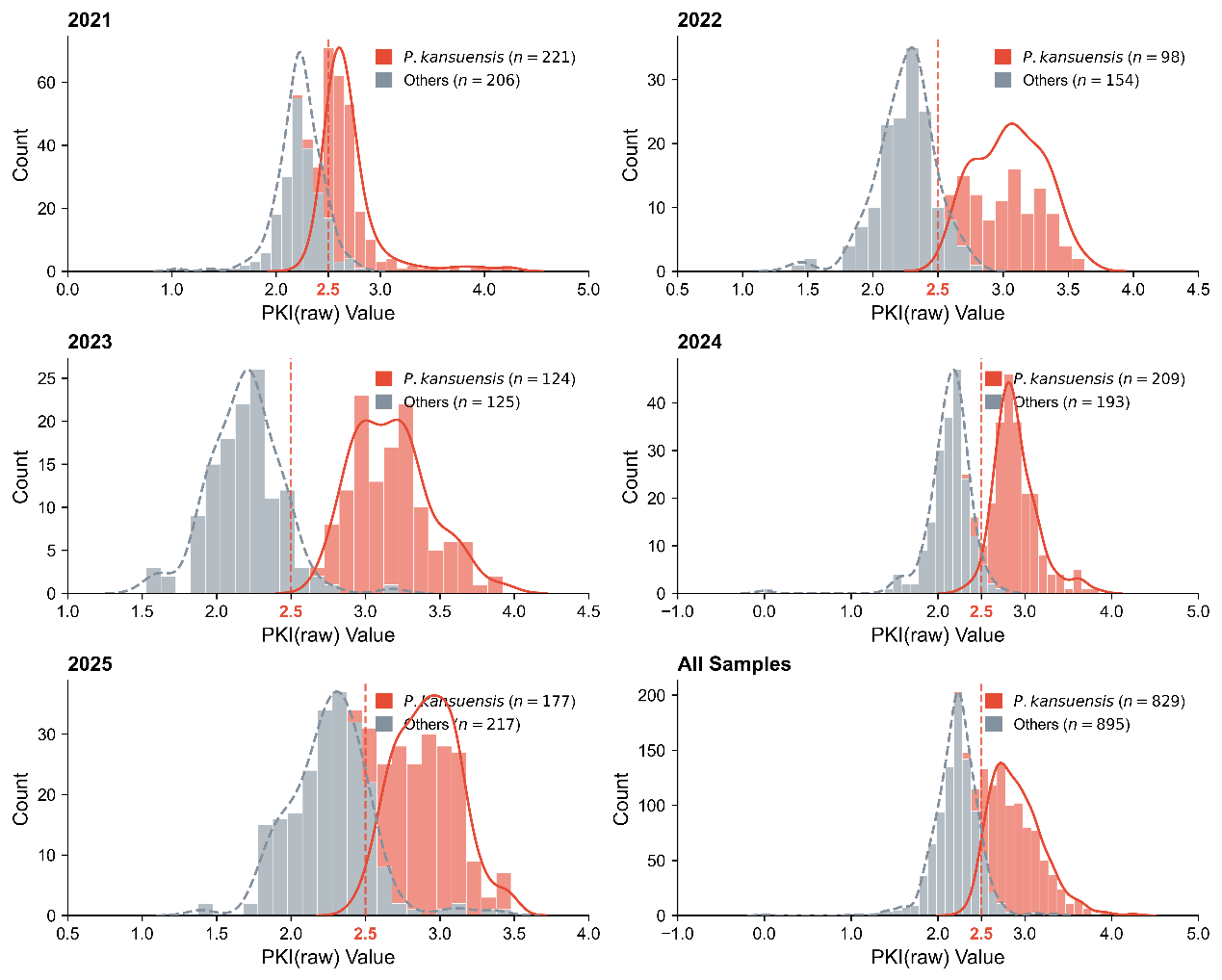


**Fig. S1.** Unscaled PKI distribution (pixels of P. kansuensis and background vegetation).

# **S2. The kernel size of GrMO**

In this paper, we use GrMO to suppress high-frequency noise in background pixels to reduce false positives. The kernel size used is 3×3 because 3×3 prevents GrMO from suppressing the target pixels. we tested 3×3, 5×5, and 7×7 kernels and evaluated their effects on PKI. The results show that overly large kernels reduce the PKI values of *P. kansuensis* pixels and thus lower the classification accuracy. Specifically, the validation accuracy was 88.11% with a 5×5 kernel and 81.78% with a 7×7 kernel. Compared with the case without GrMO (91.34%), the 3×3 kernel provided the best improvement, increasing the validation accuracy to 93.52%.


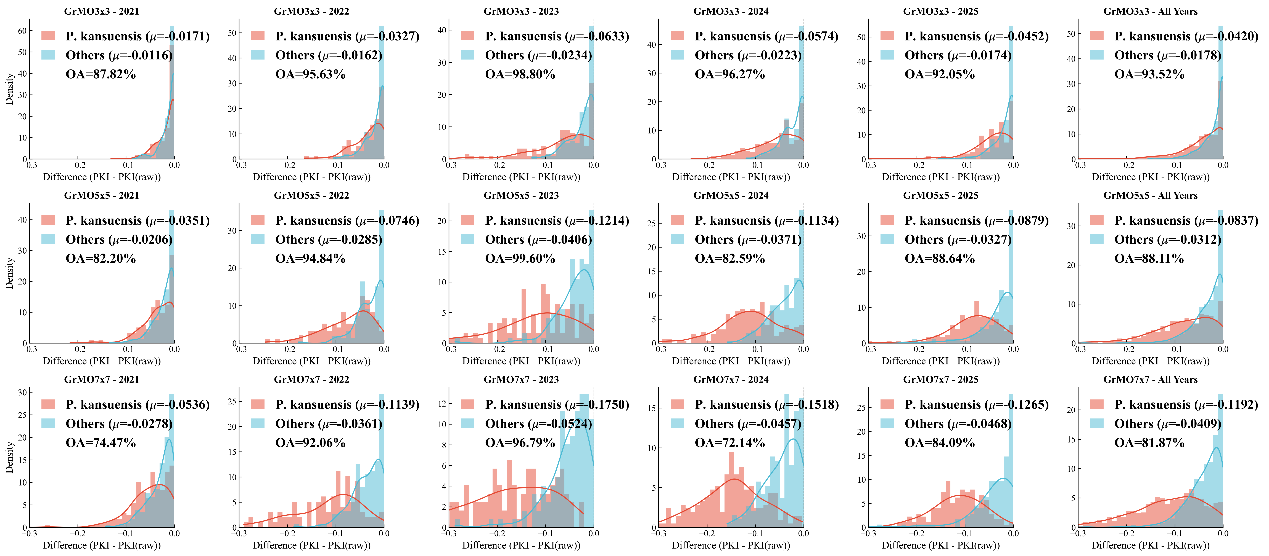


**Fig. S2.** The performance of GrMO with different kernel size

# **S3. Whisker-based thresholding**

Let $X=\{x_{i}{\}}_{i=1}^{n}$be the set of valid observations, with $n\geq5$.

**Quartiles and interquartile range:**

| $Q_{1}=P_{25}(X)$ | (1) |
| --- | --- |
| $Q_{3}=P_{75}(X)$ | (2) |
| $\mathrm{IQR}=Q_{3}-Q_{1}$ | (3) |

where $P_{p}(X)$denotes the $p$-th percentile of $X$.

**Fences (outlier cutoffs)**

| $L=Q_{1}-1.5\text{ }\mathrm{IQR}$ | (4) |
| --- | --- |
| $U=Q_{3}+1.5\text{ }\mathrm{IQR}$ | (5) |

**In-range subset:**

| $X^{'}=\{x\in X\mid L\leq x\leq U\}$ | (6) |
| --- | --- |

**Lower and upper whiskers:**

If $X^{'}\neq\emptyset$, the whiskers are defined as:

| $W_{\text{low}}=\min(X^{'})$ | (7) |
| --- | --- |
| $W_{\text{up}}=\max(X^{'})$ | (8) |

If $X^{'}=\emptyset$(rare case), the fences are returned as whiskers:

| $W_{\text{low}}=L$ | (9) |
| --- | --- |
| $W_{\text{up}}=U$ | (10) |

# **S4. Quantitative analysis for band selection**

In this study, PKI was constructed using the Blue, Green, and Red-edge bands. To quantitatively evaluate the separability of different PlanetScope bands for discriminate P. kansuensis from background pixels, we computed the Jeffries–Matusita (JM) distance for each band. The results show that the **Red** and Red-edge bands provide the highest separability, followed by **Coastal Blue** and **Blue**. We selected Red-edge rather than Red because it performs better in areas with exposed soil and helps reduce confusion in mixed soil–vegetation conditions. We selected **Blue** rather than Coastal Blue because the Coastal Blue band contains many zero-value pixels in practice, which can reduce stability.

Although the Green band shows relatively lower separability, it serves well as a normalization term in the PKI formulation. In addition, *P. kansuensis* tends to have lower reflectance in the Green band than typical background vegetation. Using Green as the denominator helps scale the index and reduces the influence of overall brightness variations and other disturbances.


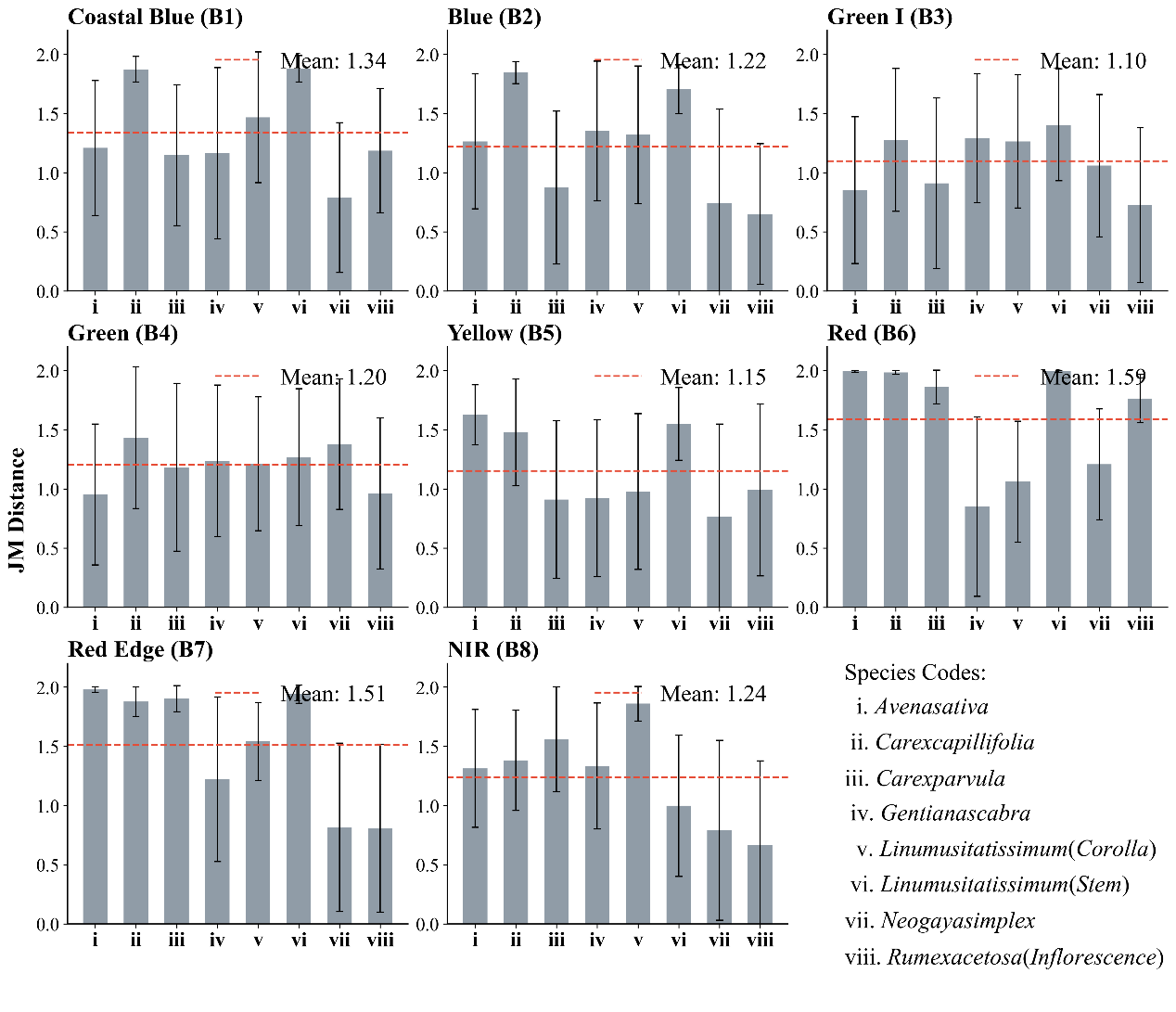


**Fig. S3.** The Jeffries–Matusita (JM) distance of P. kansuensis and background pixels at each PlanetScope band
